# HSPA12A is required for adipocyte differentiation and diet-induced obesity through a positive feedback regulation with PPARγ

**DOI:** 10.1038/s41418-019-0300-2

**Published:** 2019-02-11

**Authors:** Xiaojin Zhang, Xuan Chen, Tao qi, Qiuyue Kong, Hao Cheng, Xiaofei Cao, Yuehua Li, Chuanfu Li, Li Liu, Zhengnian Ding

**Affiliations:** 1 Department of Geriatrics, Jiangsu Provincial Key Laboratory of Geriatrics,Key Laboratory of Targeted Intervention of Cardiovascular Disease, The First Affiliated Hospital with Nanjing Medical University, 210029 Nanjing, China; 2 Department of Anesthesiology, The First Affiliated Hospital with Nanjing Medical University, 210029 Nanjing, China; 3 Key Laboratory of Targeted Intervention of Cardiovascular Disease, Collaborative Innovation Center for Cardiovascular Disease Translational Medicine, Nanjing Medical University, 210029 Nanjing, China; 4 Department of Surgery, East Tennessee State University, Johnson City, TN 37614 USA

**Keywords:** Experimental models of disease, Endocrine system and metabolic diseases

## Abstract

Obesity is one of the most serious public health problems. Peroxisome proliferator-activated receptor γ (PPARγ) plays the master role in adipocyte differentiation for obesity development. However, optimum anti-obesity drug has yet been developed, mandating more investigation to identify novel regulator in obesity pathogenesis. Heat shock protein 12A (HSPA12A) encodes a novel member of the HSP70 family. Here, we report that obese patients showed increased adipose HSPA12A expression, which was positively correlated with increase of body mass index. Intriguingly, knockout of HSPA12A (*Hspa12a*^*−/−*^) in mice attenuated high-fat diet (HFD)-induced weight gain, adiposity, hyperlipidemia, and hyperglycemia compared to their wild type (WT) littermates. Increased insulin sensitivity was observed in *Hspa12a*^−/−^ mice compared to WT mice. The HFD-induced upregulation of PPARγ and its target adipogenic genes in white adipose tissues (WAT) of *Hspa12a*^*−/−*^ mice were also attenuated. Loss- and gain-of-function studies revealed that the differentiation of primary adipocyte precursors, as well as the expression of PPARγ and target adipogenic genes during the differentiation, was suppressed by HSPA12A deficiency whereas promoted by HSPA12A overexpression. Importantly, PPARγ inhibition by GW9662 reversed the HSPA12A-mediated adipocyte differentiation. On the other hand, HSPA12A expression was downregulated by PPARγ inhibition but upregulated by PPARγ activation in primary adipocytes. A direct binding of PPARγ to the PPAR response element in the *Hspa12a* promoter region was confirmed by chromatin immunoprecipitation assay, and this binding was increased after differentiation of primary adipocytes. These findings indicate that HSPA12A is a novel regulator of adipocyte differentiation and diet-induced obesity through a positive feedback regulation with PPARγ. HSPA12A inhibition might represent a viable strategy for the management of obesity in humans.

## Introduction

Obesity, a major worldwide epidemic, is characterized by excessive accumulation of white adipose tissue (WAT), resulting from both hypertrophy of pre-existing adipocytes and differentiation of adipocyte precursors into mature adipocytes [1, 2]. Adipose tissue was considered to be purely a form of connective tissue 80 years ago, but is now known to be an important endocrine organ, lying at the center of energy homeostasis [3]. Indeed, mounting evidence demonstrates that obesity is closely correlated with abnormalities in important physiological parameters, such as lipidemia and insulin sensitivity, and is an independent risk factor for stroke, myocardial infarction, type II diabetes, and certain cancers [4]. Unfortunately, no ideal anti-obesity drug has yet been developed [5], suggesting that a more comprehensive understanding of the mechanisms underlying the development of obesity is urgently required to facilitate the development of more effective targeted therapies.

Peroxisome proliferator-activated receptor γ (PPARγ), a member of the nuclear receptor superfamily of ligand-dependent transcription factors, is considered to be a master regulator of adipocyte differentiation [4, 6–9]. Numerous studies have shown that adipogenesis involves the activation of two waves of transcription factors. The first is transiently induced by adipogenic stimuli and involves CCAAT enhancer-binding proteins (C/EBP) β and -ε, which, in turn, directly induce expression of the second wave, consisting of PPARγ and C/EBPα [6, 10]. Subsequently, PPARγ and C/EBPα positively feeds back to amplify their own expression, and these transcription factors are integral to the activation of the downstream target genes that initiate the adipogenic program. However, although it works in concert with C/EBPα, PPARγ expression is necessary and sufficient for adipogenesis [8]. In support of this, inhibition of PPARγ by antagonists ameliorates high-fat diet (HFD)-induced obesity and impairments of glucose and lipid homeostasis [8, 11]. Moreover, dominant-negative PPARγ mutation in humans and PPARγ deficiency in mice lead to lipodystrophy, and PPARγ-deficient embryonic stem cells are unable to differentiate into adipocytes [8, 12]. Therefore, targeting PPARγ may represent a promising approach for the management of obesity.

Heat shock proteins (HSPs) are an evolutionarily conserved superfamily of protein chaperones that exhibit diverse functions, such as the facilitation of protein folding, translocation, trafficking, and the targeted removal of aberrant proteins [13, 14]. Several HSPs, including HSP90, HSPA5 (GRP78), and DNAJB1 (HSP40), are involved in adipogenesis; for example, HSP90 promotes and DNAJB1 suppresses adipocyte differentiation [8, 15–18]. Heat shock protein A12A (HSPA12A), which was cloned from mouse atherosclerotic lesions in 2003, is a novel and distinct member of the mammalian heat shock protein 70 (HSP70/HSPA) family due to it containing an atypical Hsp70 ATPase domain [19, 20]. Subsequent studies have shown that *Hspa12a* mRNA is expressed at a high level in human brain, and its cerebral expression was decreased in the patients with schizophrenia [19, 21, 22]. Recently, we reported that HSPA12A encodes a novel survival pathway that protects against ischemic stroke in mice [23]. However, the functional roles of HSPA12A in adipose tissue remain to be investigated.

In this study, obese patients showed increased HSPA12A expression in WAT, while deficiency of HSPA12A in mice ameliorated HFD-induced obesity, hyperlipidemia and hyperglycemia. Studies of loss-of-function and gain-of-function demonstrated that HSPA12A was required for adipocyte differentiation via maintaining PPARγ expression. Inversely, we also identified the regulation of PPARγ in HSPA12A expression by directly binding to the peroxisome proliferator response element (PPRE) in the *Hspa12a* promoter. Our findings imply that HSPA12A is a novel regulator of adipocyte differentiation and diet-induced obesity through a positive feedback regulation with PPARγ. Thus, HSPA12A inhibition might represent a viable strategy for the therapy of obesity in humans.

## Results

### HSPA12A is highly expressed in murine adipose tissues

**Fig. 1:**
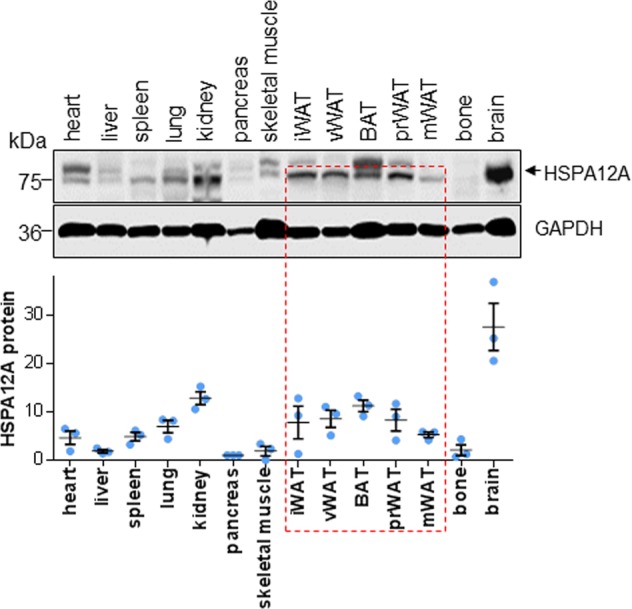
HSPA12A expressed at high level in adipose tissues. Fourteen types of tissues including adipose tissues were collected from adult C57BL/6 mice. Protein extracts were prepared for immunoblotting against HSPA12A. Blots against GAPDH served as loading controls. *n* = 3/group

The expression profile of HSPA12A in adipose tissues has not been characterized. Immunoblotting revealed a high level of HSPA12A expression in adipose tissues, including inguinal WAT (iWAT), visceral WAT (vWAT), peri-renal WAT (prWAR), and brown adipose tissue (BAT). Although lower than in brain, HSPA12A expression was much higher in adipose tissue than in other tissues, such as heart, liver, pancreas, spleen, lung, skeletal muscle, and bone (Fig. 1).

### HSPA12A expression is positively correlated with body mass index in humans

**Fig. 2:**
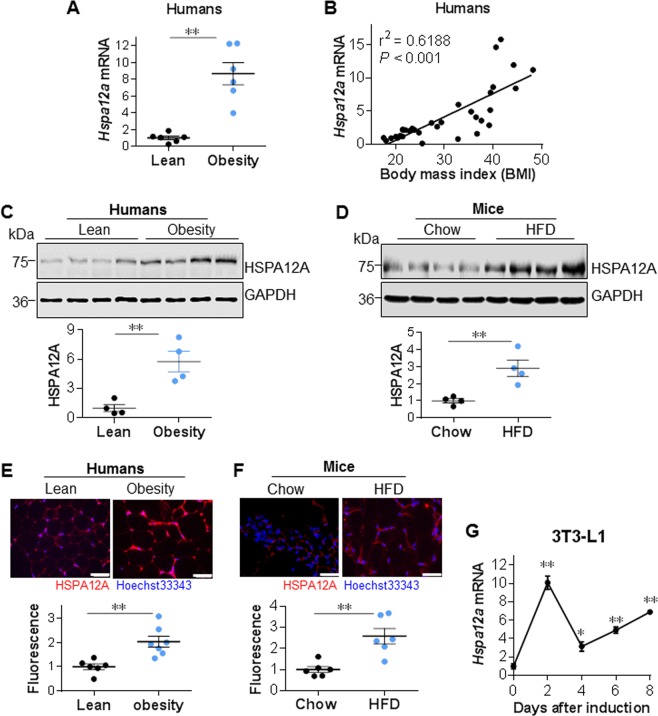
HSPA12A upregulation was positively correlated with the increase of human BMI. **a** *Hspa12a* mRNA expression was examined in sWAT of obese patients (BMI > 35) and lean subjects (BMI < 24) using real-time PCR. Data are mean ± SEM, **P *<* 0.01 by Student’s two-tailed unpaired *t-*test. *n* = 6 human subjects/group. **b** *Hspa12a* mRNA was examined in sWAT of humans. Linear regression was analyzed. **c** HSPA12A protein expression was examined in human sWAT using immunoblotting. Blots against GAPDH served as loading controls. Data are mean ± SEM, **P *<* 0.01 by Student’s two-tailed unpaired *t-*test. *n* = 4 subjects/groups. **d** HSPA12A protein expression was examined in iWAT of HFD-induced obese mice and chow-fed control mice. Blots against GAPDH served as loading controls. Data are mean ± SEM, **P *<* 0.01 by Student’s two-tailed unpaired *t-*test. *n* = 4 mice/groups. **e** Immunofluorescence staining for HSPA12A was examined on cryosections of human sWAT. Hoechst 33342 was used to counterstain nuclei. Fluorescence intensity of HSPA12A staining was quantified and expressed as mean ± SEM. **P *<* 0.01 by Student’s two-tailed unpaired *t-*test. *n* = 6–7 subjects/group. Scale bar = 50 μm. **f** Inguinal WAT were collected from obese mice that induced by HFD. Chow diet-fed mice served as controls. Cryosections were prepared for immunofluorescence staining against HSPA12A. Hoechst 33342 was used to counterstain nuclei. Fluorescence intensity of HSPA12A staining was quantified and expressed as mean ± SEM. **P *<* 0.01 by Student’s two-tailed unpaired *t-*test. *n* = 6 mice/group. Scale bar = 50 μm. **g** *Hspa12a* mRNA was examined in 3T3-L1 cells at the indicated time points after differentiation induction. Data are mean ± SEM, **P *<* 0.01 or *P *<* 0.05 vs. untreated controls (0 day) by One-way ANOVA followed by Tukey’s test. *n* = 3 cultures/group

Identification of the high expression level of HSPA12A in WAT prompted us to investigate its clinical significance in adipogenesis. Obese patients showed markedly higher expression of HSPA12A at both mRNA and protein levels in subcutaneous WAT (sWAT) than lean subjects (Fig. 2a–c and S1A). Notably, upregulation of HSPA12A positively correlated with the increase of body mass index (BMI) (Fig. 2b). Consistent with this, HFD-induced obese mice also showed significantly higher HSPA12A expression in iWAT than chow-fed mice did (Fig. 2d and S1B, S2).

Increased HSPA12A expression was visualized in adipocytes of obese humans and mice using immunofluorescence staining (Fig. 2e, f). In addition, a markedly upregulation of HSPA12A expression was detected in 3T3-L1 preadipocytes after differentiation induction (Fig. 2g). Thus, HSPA12A expression was increased in WAT adipocytes during their differentiation.

### HSPA12A deficiency ameliorates high-fat diet-induced weight gain and adiposity in mice

**Fig. 3:**
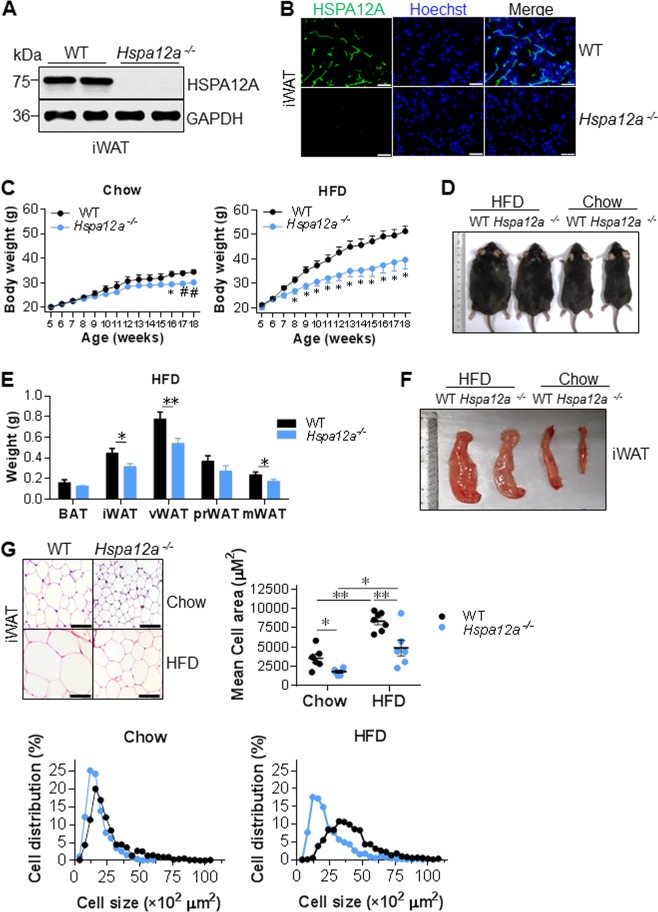
Deficiency of HSPA12A attenuated the HFD-induced weight gain and adiposity. **a**, **b** HSPA12A expression. Inguinal WAT were collected from adult mice. HSPA12A expression was analyzed by immunoblotting (**a**) and immunofluorescence staining (**b**, Scale bar = 20 μm). Note that HSPA12A expression was absent in *Hspa12a*^*-/-*^ mice. *n* = 10 mice/group. WT, wild type; *Hspa12a*^*-/-*^, HSPA12A knockout. **c**, **d** Body weights. Mice aged at 5-week old were fed with HFD or normal chow diet for 14 weeks. Body weight was recorded weekly (**c**). The representative mice size at the end of experiments was also shown (**d**). Data are mean ± SEM, *P *<* 0.01 and ^#^*P* *<* 0.05 vs. the age-matched WT mice, two-way ANOVA followed by Tukey’s test. *n* = 4–6 mice/Chow group and *n* = 6–8 mice /HFD group. **e**, **f** Adipose weights. Weights of the indicated adipose tissues were measured in the mice fed with HFD for 14 weeks (**e**). The representative images of iWAT were also shown (**f**). Data are mean ± SEM, **P *<* 0.01 and *P *<* 0.05 by Student’s two-tailed unpaired *t-*test. *n* = 27 mice /group. BAT, brown adipose tissue; iWAT, inguinal WAT; vWAT, visceral WAT; prWAT, peri-renal WAT; mWAT, mesentery WAT. **g** Adipocyte areas. H&E staining was performed on the paraffin-embedded sections of iWAT from mice fed with HFD or chow diet for 14 weeks. Adipocyte areas were measured. Data are mean ± SEM, **P *<* 0.01 and *P *<* 0.05 by two-way ANOVA followed by Tukey’s test. *n* = 6–7 mice/group. Scale bar = 50 μm

To investigate whether HSPA12A is required during adipogenesis, we generated HSPA12A knockout mice (*Hspa12a*^−/−^) using the Cre-*lox*P recombination system [23]. The successful deletion of the *Hspa12a* gene in WAT was confirmed by the absence of HSPA12A protein expression, as indicated by both immunoblotting and immunostaining (Fig. 3a, b and S3).

After being fed a HFD for 14 weeks, *Hspa12a*^−/−^ mice showed less body mass gain than WT mice (Fig. 3c, d). Accordingly, *Hspa12a*^−/−^ mice had smaller iWAT, vWAT, and mesenteric WAT (mWAT) depots than WT controls after HFD feeding (Fig. 3e, f). Consistent with this, *Hspa12a*^−/−^ mice showed smaller adipocytes of iWAT, both in terms of mean cell size and cell size distribution, than WT controls after HFD feeding (Fig. 3g). In addition, in the chow-fed groups, *Hspa12a*^−/−^ mice showed less body mass gain and smaller iWAT adipocytes than WT controls at 16 weeks of age, although no differences in body length or food intake were observed between the two genotypes (Fig. 3c–g, and S4A–B).

### HSPA12A deficiency attenuates the high-fat diet-induced abnormalities in serum metabolic parameters

**Fig. 4:**
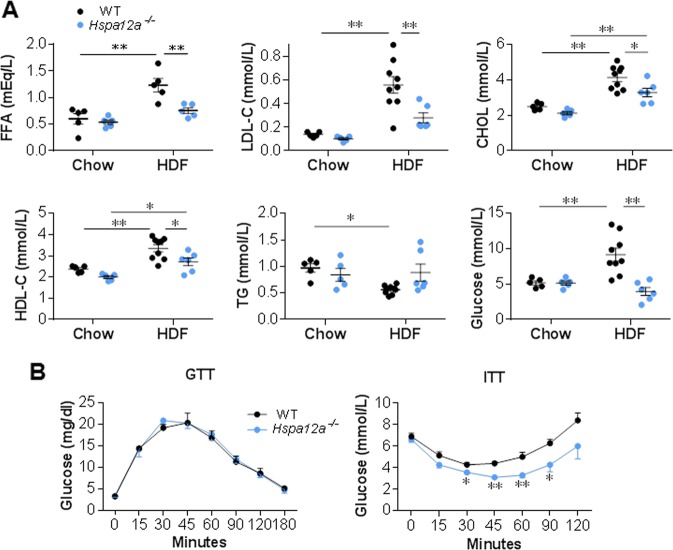
Deficiency of HSPA12A attenuated the HFD-induced hyperlipemia and hyperglycemia. **a** Blood metabolic parameters. Fasting serum samples were collected from mice fed with HFD or chow diet for 14 weeks for measurements of the indicated parameters. Data are mean ± SEM, **P *<* 0.01 and *P *<* 0.05 by two-way ANOVA followed by Tukey’s test. *n* = 5–9 mice/group. **b** GTT and ITT were performed in 18-week old mice after fasting for 16 h and 4 h, respectively. Data are mean ± SEM, **P *<* 0.01 and *P *<* 0.05 vs. the time-matched WT mice by two-way ANOVA followed by Tukey’s test. *n* = 4 mice/GTT group and *n* = 7 mice/ITT group

High serum levels of free fatty acid (FFA), cholesterol (CHOL), low-density lipoprotein cholesterol (LDL-C), and glucose are commonly observed in obesity [24]. As shown in Fig. 4a, the HFD-induced increases in serum FFA, LDL-C, CHOL, high-density lipoprotein cholesterol (HDL), and glucose were attenuated in *Hspa12a*^−/−^ mice compared with WT controls. Also, lower serum FFA content was detected in *Hspa12a*^−/−^ mice than in WT controls when both were fed a HFD.

### Increased insulin sensitivity in *Hspa12a*^−/−^ mice

Changes in adipose tissue mass are frequently associated with alterations in glucose homeostasis [25]. We therefore carried out insulin and glucose tolerance tests (ITT, GTT) to determine whether insulin and glucose homeostasis are affected by *Hspa12a* knockout. Although GTT demonstrated no difference in glucose concentration or clearance rate between the two genotypes, ITT showed that *Hspa12a*^−/−^ mice have significantly increased insulin sensitivity (Fig. 4b).

### *Hspa12a*^−/−^ mice demonstrate attenuated expression of PPARγ and its target genes linking to adipogenesis upon high-fat diet feeding

**Fig. 5:**
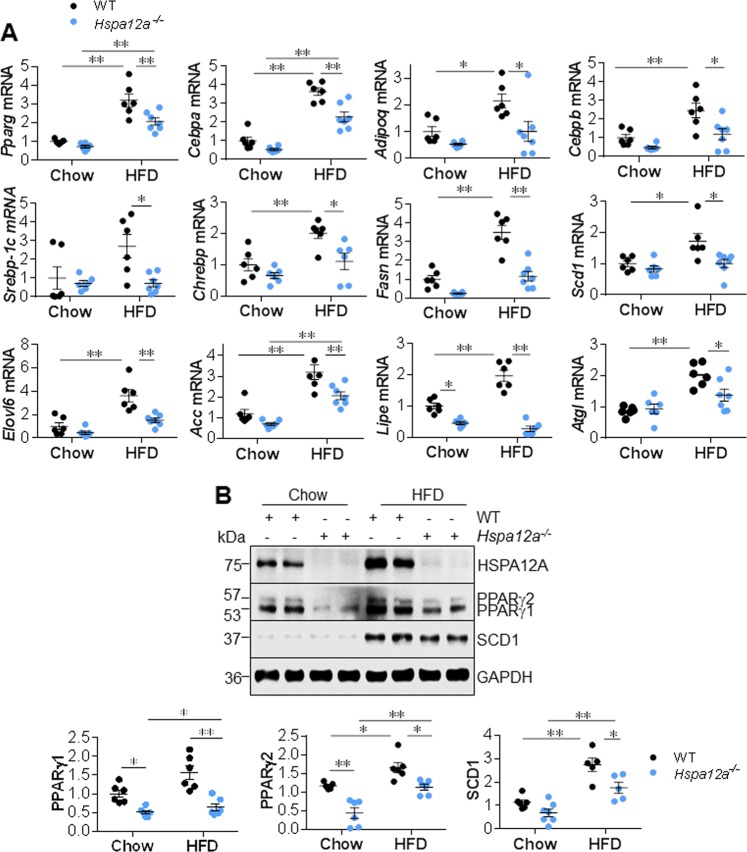
Deficiency of HSPA12A suppressed PPARγ and its target genes linking to adipocyte differentiation in mice. Inguinal WAT were collected from mice that fed with HFD or chow diet for 14 weeks. The expression of the indicated mRNA and proteins was analyzed by Real-time PCR (**a**) and Immunoblotting (**b**), respectively. Data are mean ± SEM, **P *<* 0.01 and *P *<* 0.05 by two-way ANOVA followed by Tukey’s test. *n* = 5–7 mice/group

PPARγ-dependent signaling plays a central role in adipogenesis [4, 6]. The HFD-induced upregulation of *Pparg* mRNA and that of its target gene *Cebpa* was attenuated in the iWAT of *Hspa12a*^−/−^ mice compared with WT controls (Fig. 5a). In addition, the HFD-induced upregulation of *Ebpb* (a regulator of the early phase of differentiation) and *Adipoq* (a marker of mature adipocytes) in iWAT was prevented in *Hspa12a*^−/−^ mice. These findings suggest that HSPA12A regulates both early and late events in adipocyte differentiation.

Next, the expression of genes involved in lipid metabolism was quantified. When HFD-fed, the iWAT of *Hspa12a*^−/−^ mice showed lower expression of the lipogenic transcription factors *Shrebp-1c* and *Chrebp* than that of WT controls (Fig. 5a). In agreement with this, their target genes that are involved in lipogenesis, including *Fasn*, *Scd1*, *Elovl6*, and *Acc*, showed the same expression profile. In addition, the HFD-induced upregulation of lipase expression (*Lipe* and *Atgl*) was prevented in *Hspa12a*^−/−^ iWAT (Fig. 5a and S5).

We next measured the protein levels of PPARγ as a representative transcription factor, and SCD1, FABP4, ACC, and C/EBPα as representative adipogenic proteins, in iWAT of mice. Consistent with the mRNA data, *Hspa12a*^−/−^ iWAT showed lower PPARγ1/2 protein expression than WT iWAT when they had been either HFD-fed or chow-fed (Fig. 5b). In addition, lower protein expression of SCD1, FABP4, C/EBPα, and ACC was found in *Hspa12a*^−/−^ iWAT than in WT iWAT, after HFD feeding. Moreover, the HFD-induced upregulation of PPARγ1/2, SCD1, FABP4, C/EBPα, and ACC, protein levels was attenuated in *Hspa12a*^−/−^ iWAT *versus* iWAT from WT controls (Fig. 5b and S6).

### HSPA12A regulates adipocyte differentiation and PPARγ expression in vitro

**Fig. 6:**
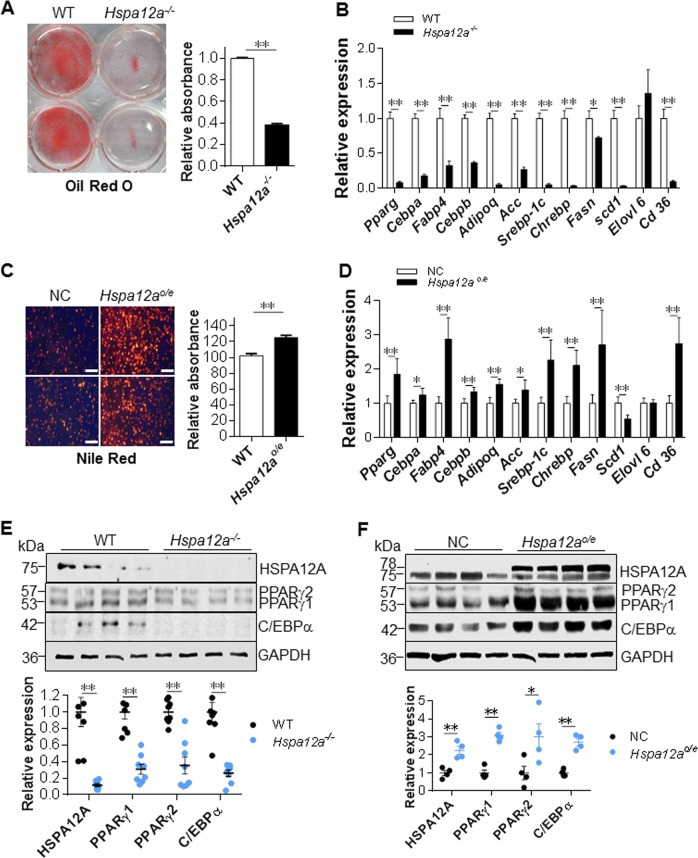
HSPA12A regulated adipocyte differentiation and PPARγ expression in vitro. **a**, **b** HSPA12A deficiency suppressed adipocyte differentiation. Differentiation was induced in primary SVF isolated from WT and *Hspa12a*^*-/-*^ mice. Lipid droplets were examined by ORO staining (**a**). Expression of mRNA was examined using real-time PCR (**b**). Data are mean ± SEM, **P *<* 0.01 and *P *<* 0.05 by Student’s two-tailed unpaired *t-*test. *n* = 11/group (ORO) and *n* = 6/group (PCR). **c**, **d** HSPA12A overexpression promoted adipocyte differentiation. Primary SVF was isolated from WT mice and was overexpressed with HSPA12A (*Hspa12a*^*o/e*^) by infection with adenovirus-carried *Hspa12a* expression sequence. The SVF infected with empty virus served as normal controls (NC). Six days after differentiation induction, lipid accumulation was evaluated by Nile red staining (**c** Scale bar = 100 μm) and mRNA levels were examined using real-time PCR (**d**). Data are mean ± SEM, **P *<* 0.01 and *P *<* 0.05 by Student’s two-tailed unpaired *t-*test. *n* = 8/group (Nile red) and *n* = 5–6/group (PCR). **e** HSPA12A deficiency decreased PPARγ expression. Primary SVF were isolated from WT and *Hspa12a*^*-/-*^ mice. Six days after differentiation, expression of the indicated proteins was examined by immunoblotting. Data are mean ± SEM, **P *<* 0.01 by Student’s two-tailed unpaired *t-*test. *n* = 8/group. **f** HSPA12A overexpression increased PPARγ expression. Primary SVF was isolated from WT mice and was overexpressed with HSPA12A (*Hspa12a*^*o/e*^). The NC SVF served as controls. Six days after differentiation induction, the indicated protein expression was examined using immunoblotting. Data are mean ± SEM, **P *<* 0.01 and *P *<* 0.05 by Student’s two-tailed unpaired *t-*test. *n* = 4/group. Note: Endogenous HSPA12A is 75 kDa, exogenous HSPA12A is 78 kDa containing 3 flags

To further investigate the regulation of HSPA12A in adipogenesis, we compared the in vitro differentiation of primary adipocyte precursors from the isolated stromal vascular fraction (SVF) between two murine genotypes. *Hspa12a*^−/−^ SVF clearly exhibited poorer differentiation, demonstrated by less staining with oil red O (ORO) and lower expression of mature adipocyte markers (*Pparg*, *Fabp4*, *Adipoq*, *Fasn*, and *Acc)* and other genes involved in differentiation (*Cebpa*, *Cebpb*, *Scebp-1c*, *Chrebp*, *Scd1,* and *Cd36*) after 6 days of differentiation, compared with WT SVF (Fig. 6a, b). By striking contrast, HSPA12A overexpressing (*Hspa12a*^*o/e*^) SVF showed enhanced differentiation, as reflected in greater Nile red staining and mRNA expression of *Pparg*, *Cebpa*, *Fabp4*, *Cebpb*, *Adipoq*, *Acc*, *Scebp-1c*, *Chrebp*, *Fasn*, and *Cd36* (Fig. 6c, d). Consistent with this, the protein levels of PPARγ1/2, C/EBPα, FABP4, ACC and SCD1 in SVF after differentiation were decreased by HSPA12A deficiency but increased by HSPA12A overexpression (Fig. 6e, f, S7A-B). When taken into account that *Scd1* mRNA levels were reduced in *Hspa12a*^*o/e*^ SVF (Fig. 6d), the data suggests that HSPA12A may also regulate SCD1 expression at post-transcriptional level.

### Inhibition of PPARγ attenuates HSPA12A-induced adipocyte differentiation

**Fig. 7:**
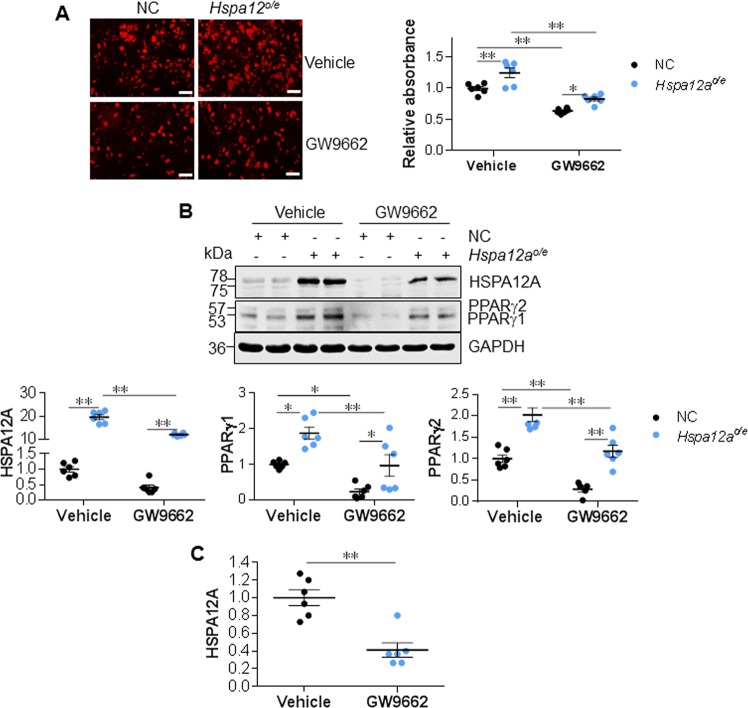
PPARγ inhibition suppressed the HSPA12A-induced adipocyte differentiation. 3T3-L1 preadipocytes were overexpressed with HSPA12A (*Hspa12a*^*o/e*^) by infected with adenovirus-carried *Hspa12a* expression sequence. 3T3-L1 preadipocytes infected with empty virus served as normal controls (NC). Six days after differentiation induction in the presence or absence of GW9662, the following experiments were performed. **a** Nile red staining was performed to evaluate lipid accumulation. Data are mean ± SEM, **P *<* 0.01 and *P *<* 0.05 by two-way ANOVA followed by Tukey’s test. *n* = 6/group. Scale bar = 100μm. **b** Protein expression was examined using immunoblotting. Data are mean ± SEM, **P *<* 0.01 and *P *<* 0.05 by two-way ANOVA followed by Tukey’s test. *n* = 5–6/group. Note: Endogenous HSPA12A is 75 kDa, exogenous HSPA12A is 78 kDa containing three flags. **c** The effect of GW9662 on HSPA12A expression in normal control 3T3-L1 cells was quantified from the blots in Fig. 7b. Data are mean ± SEM, **P *<* 0.01 by Student’s two-tailed unpaired *t-*test. *n* = 6/group

PPARγ expression was reduced by HSPA12A deficiency but increased by HSPA12A overexpression during adipocyte differentiation in both in vivo and in vitro models (Figs. 5–6). To elucidate whether PPARγ mediates the regulation of adipogenesis by HSPA12A, we followed the differentiation of normal control (NC) and HSPA12A*-*overexpressing (*Hspa12a*^*o/e*^) 3T3-L1 cells in the presence or absence of a potent specific PPARγ inhibitor (GW9662) [26]. Similar to the observations made in primary SVF (Fig. 6c), HSPA12A overexpression enhanced 3T3-L1 differentiation, demonstrated by greater Nile red staining after 6 days of differentiation (Fig. 7a). In addition, HSPA12A overexpression increased PPARγ1/2 expression in differentiated 3T3-L1 adipocytes (Fig. 7b). Importantly, PPARγ inhibition with GW9662 suppressed the differentiation of *Hspa12a*^*o/e*^ 3T3-L1 cells, demonstrated by less Nile red staining than that in *Hspa12a*^*o/e*^ cells without GW9662 treatment (Fig. 7a).

### *Hspa12a* is a novel target gene of PPARγ

**Fig. 8:**
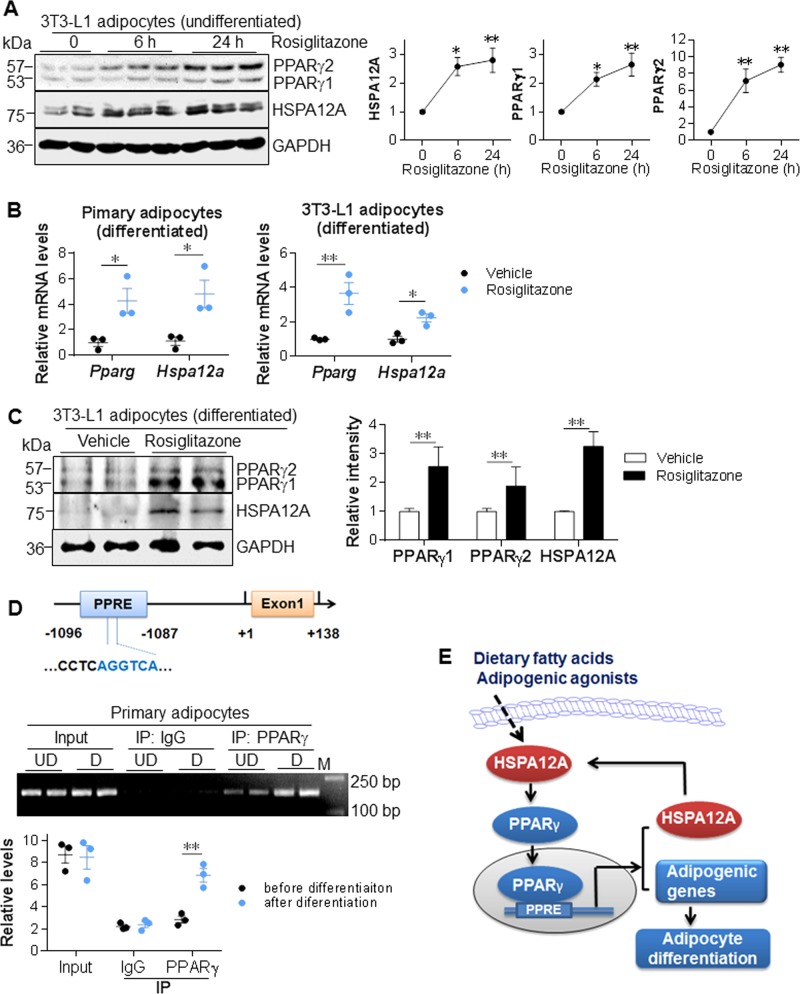
HSPA12A is a novel target gene of PPARγ in adipocytes. **a** PPARγ activation increased HSPA12A expression in undifferentiated preadipocytes. 3T3-L1 preadipocytes were treated with rosiglitazone for the indicated times. HSPA12A protein levels were examined by immunoblotting. Data are mean ± SEM, **P *<* 0.01 and *P *<* 0.05 vs. 0 h controls by one-way ANOVA followed by Tukey’s test. *n* = 5–8/group. **b**, **c** PPARγ activation increased HSPA12A expression in differentiated adipocytes. Rosiglitazone was administrated to primary SVF and 3T3-L1 adipocyte cultures for six days since differentiation induction. Levels of mRNA and protein expression were examined by real-time PCR (**b**) and immunoblotting protein (**c**). Data are mean ± SEM, **P *<* 0.01 and *P *<* 0.05 by Student’s two-tailed unpaired *t-*test. *n* = 3/group (PCR) and *n* = 5–7/group (immunoblotting). **d** ChIP analysis. Primary SVF with or without differentiation were collected for ChIP analysis to examine whether PPARγ binds to the PPRE site in *Hspa12a* promoter. Input and IgG-immunoprecipitated samples served as positive and negative controls, respectively. Data were collected by real-time PCR assay (down panel). The PCR products were also visualized by running agarose gel (middle panel). Data are mean ± SEM, ** *P* *<* 0.01 by Student’s two-tailed unpaired *t-*test. *n* = 3/group. **e** Mechanism scheme. HSPA12A is upregulated by adipogenic agonists in preadipocyte precursors. HSPA12A promotes PPARγ expression, which in turn activates HSPA12A expression through binding to the PPRE in *Hspa12a* promoter as well as activates a group of other adipogenic genes to initiate adipocyte differentiation. Therefore, HSPA12A promotes adipogenesis through a positive feedback regulation loop with PPARγ

Unexpectedly, lower HSPA12A expression at both protein and mRNA levels was found following PPARγ inhibition (Fig. 7b, c, S8), suggesting a regulatory effect of PPARγ on HSPA12A expression. To further investigate this, we determined whether HSPA12A expression would be increased by the PPARγ activator rosiglitazone, and indeed, rosiglitazone treatment upregulated HSPA12A expression in undifferentiated 3T3-L1 cells in a time-dependent manner (Fig. 8a). Moreover, rosiglitazone upregulated HSPA12A expression at the mRNA and protein levels in both primary SVF and 3T3-L1 adipocytes after differentiation (Fig. 8b, c). Taken together, these data indicate that HSPA12A expression is regulated by PPARγ.

Gene promoter analysis (http://gene-regulation.com) showed the presence of one putative PPRE site within the *Hspa12a* promoter at position −1096/−1087 (upper panel of Fig. 8d). To determine whether PPARγ binds to the *Hspa12a* promoter at this site, a Chromatin immunoprecipitation (ChIP) assay was performed. This confirmed the binding of PPARγ to the PPRE in the *Hspa12a* promoter in primary SVF cells, and this binding was significantly greater in differentiated SVF than in undifferentiated control SVF (lower panel of Fig. 8d).

### No direct protein interaction between PPARγ and HSPA12A

PPARγ protein level has been shown to be increased by HSP90 through the formation of a HSP90-PPARγ complex to prevent PPARγ degradation [8, 27]. To investigate whether HSPA12A increased PPARγ protein level in the similar way as HSP90 doing, we performed immunoprecipitation-western blot analysis. The 3T3-L1 cells with or without differentiation were precipitated with anti-HSPA12A antibody. The cell lysates without immunoprecipitation served as positive controls (input), while the immunoprecipitates with normal IgG served as negative controls. As shown in Figure S9, PPARγ protein was not recovered in the HSPA12A immunocomplexes.

## Discussion

Our study uncovers a requirement for HSPA12A in adipocyte differentiation and HFD-induced obesity. This action of HSPA12A was in a PPARγ-depended manner. We have also identified a positive feedback regulation between HSAP12A and PPARγ in adipocytes. HSPA12A inhibitory strategy might represent a novel therapeutic approach for obese patients.

Heat shock proteins are an evolutionarily conserved superfamily comprising a group of structurally unrelated subfamilies, including HSPA/HSP70, HSPB/HSP27, HSPC/HSP90, HSPH/HSP110, and NDAJ/HSP40 [28]. Of these, HSP90, HSPA5/GRP78, and DNAJB1/HSP40 are involved in adipogenesis [8, 15–17]. As an example, HSP90 regulates adipocyte differentiation by chaperoning PPARγ to control its stability, and HSP90 inhibition impedes the differentiation of 3T3-L1 preadipocytes and lipid accumulation [8, 18]. Moreover, HSPA5 is required for adipogenesis because when absent the differentiation of 3T3-L1 cells is impaired and mice demonstrate lipoatrophy [15]. By contrast, DNAJB1 overexpression decreases both lipid accumulation and the expression of adipocyte markers in 3T3-L1 cells, suggesting that it has an inhibitory role in adipocyte differentiation [17]. In this study, we found that HSPA12A, a novel member of the HSPA/HSP70 family, is upregulated in the WAT of obese humans, and that its expression is positively correlated with BMI, suggesting a possible involvement of HSPA12A in adipogenesis. Indeed, the differentiation of primary adipocyte precursors was suppressed by HSPA12A deficiency, whereas it was enhanced by HSPA12A overexpression. Moreover, HFD-induced and age-associated weight gain and adiposity were attenuated in HSPA12A knockout mice. Taken together, these results provide strong evidence that HSPA12A is a novel regulator of adipogenesis.

Obesity is usually associated with metabolic abnormalities, including hyperlipidemia, hyperglycemia, and insulin resistance [29], and all of these are independent risk factors for shortened lifespan and the development of atherosclerosis, myocardial infarction, stroke, and type II diabetes [29, 30]. In this study, we observed that the HFD-induced elevation of serum LDL, cholesterol, and glucose was either prevented or ameliorated in *Hspa12a*^−/−^ mice. Moreover, *Hspa12a*^−/−^ mice showed higher insulin sensitivity than control mice at 18 weeks of age. Thus, HSPA12A deficiency ameliorates HFD-induced defects in lipid and glucose homeostasis.

Adipogenesis involves a complex network of regulatory proteins, but PPARγ is its master regulator. Of its two major isoforms, PPARγ1 is expressed in various tissues, including adipose tissue, liver, macrophages, and skeletal muscle, while PPARγ2 is exclusively present in adipogenic cells, but both play critical roles in adipogenesis [8, 31]. We found that PPARγ expression was lower at both the mRNA and protein level in the WAT of *Hspa12a*^−/−^ mice than in WT controls, when both were fed with either a chow or HFD diet, suggesting a regulatory effect of HSPA12A on PPARγ expression. This finding was further confirmed by in vitro experiments, which demonstrated that the expression of PPARγ was increased by HSPA12A overexpression, whereas it was decreased by HSPA12A deficiency, in differentiated primary adipocytes, suggesting an effect of HSPA12A on PPARγ expression during adipocyte differentiation. Most importantly, inhibition of PPARγ with GW9662 reversed the HSPA12A-induced enhancement of adipocyte differentiation. Collectively, our data suggest that HSPA12A regulates adipogenesis in a PPARγ-dependent manner. Previous studies have demonstrated that PPARγ expression is regulated by HSP90 through the formation of a HSP90-PPARγ complex, which prevents the proteasomal degradation of PPARγ [8, 27]. However, we did not found PPARγ protein in HSPA12A immunocomplexes from either undifferentiated or differentiated adipocytes (Figure S9), suggesting that HSPA12A increasing PPARγ protein expression is not through the direct interaction between the two proteins. The *Pparg* mRNA expression showed increase by HSPA12A overexpression whereas decrease by HSPA12A knockout, suggesting a regulation of HSPA12A at *Pparg* transcription levels. During adipogenesis, the transcription of *Pparg* con be regulated by a group of factors, such as C/EBPα, SREBP-1, early B-cell factors, and et al. [32]. Indeed, in this study, we observed that in WAT and adipocytes, the expression of C/EBPα and SREBP-1 were both reduced by HSPA12A deficiency while increased by HSPA12A overexpression, suggesting a possible involvement of C/EBPα and SREBP-1 in the regulation of HSPA12A on *Pparg* transcription. It is worthwhile in further work to clarify the exact mechanism for how HSPA12A modulates PPARγ transcription.

Unexpectedly, lower HSPA12A expression was also observed in adipocytes following PPARγ inhibition, suggesting a possible regulatory effect of PPARγ on HSPA12A expression. This suggestion is supported by the observation that the PPARγ activator rosiglitazone increases HSPA12A expression at both the mRNA and protein levels in adipocytes either before or after differentiation. As a central regulator of adipogenesis, PPARγ directly drives the expression of a group of target genes involved in adipocyte differentiation by binding to PPREs in their promoters. Our ChIP assay confirmed the binding of PPARγ to the PPRE located at −1096/−1087 in the *Hspa12a* promoter, and this binding was increased after differentiation. These findings identify HSPA12A as a novel target gene of PPARγ in adipocytes. When combined with the regulation of PPARγ expression by HSPA12A in WAT in vivo and in adipocytes in vitro, the data collectively suggest a positive feedback regulation between HSPA12A and PPARγ in adipocytes (Fig. 8e).

In conclusion, this study demonstrates that HSPA12A is required for adipogenesis, and deficiency of HSPA12A attenuates the HFD-induced obesity and impairments of lipid and glucose. The mechanism underlying the effects of HSPA12A on adipogenesis involves a positive feedback regulation with PPARγ. Our data strongly suggest that inhibitors of HSPA12A may be useful for the management of obesity in humans.

## Materials and methods

### Reagents

Collagenase Type II, ORO, Nile red, paraformaldehyde (PFA), rosiglitazone, 3-isobutyl-1-methylxanthine (IBMX) and dexamethasone were from Sigma-Aldrich (St. Louis, MO). GW9662 was from Medchemexpress (Monmouth Junction, NJ). FFA assay kit was from Wako-chem (Osaka, Japan). Trizol reagent was from Life Technology (Carlsbad, CA). Bovine serum albumin (BSA) was from Roche (Basel, Switzerland). Normal Goat Serum was from Jackson ImmunoResearch (West Grove, PA). DMEM medium and fetal bovine serum (FBS) was from Gibco (Shelton, CT). High-sig ECL western blotting substrate was from Tanon (Shanghai, China). Protein A-Agarose was from Santa Cruz Biotechnology (Dallas, TX).

### Human samples

Abdominal subcutaneous white fat specimens and blood samples were collected from bariatric surgery patients with obesity and cholecystectomy patients with cholelithiasis in the First Affiliated Hospital of Nanjing Medical University. Patients were fasted for 12 h before blood sampling. All the recruited patients were without infection, cancer or any other ischemic disorders. Patients gave informed consent at the time of recruitment. The Ethical Board of First Affiliated Hospital of Nanjing Medical University approved this study (# 2016-SR-123), and all the human studies were conformed to the principles set out in the WMA Declaration of Helsinki and the Department of Health and Human Services Belmont Report.

### Creation of *Hspa12a* knockout mice

The *Hspa12a* targeting vector was constructed using bacterial artificial chromosome (BAC) retrieval method [16, 23, 33]. Briefly, the region of the *Hspa12a* gene containing exons 2–4 was retrieved from a 129/sv BAC clone (BAC/PAC Resources Center, Oakland, CA) using a retrieval vector containing two homologous arms. Exons 2 and 3 were replaced by *lox*P sites flanking a PGK-neo cassette as a positive selection marker (Figure S10). Embryonic stem cells were electroporated with the linearized targeting vector, selected, and then expanded for Southern blot analysis. Chimeric mice (*Hspa12a*^*flox/+*^) were generated by injecting embryonic stem cells into C57BL/6 blastocysts, followed by transferring into pseudo-pregnant mice. To remove the *Hspa12a* gene, the chimeric mice were crossed with EIIa-Cre transgenic mice.

The mice were bred at the Model Animal Research Center of Nanjing University and were maintained in the Animal Laboratory Resource Facility of the same institution. All experiments conformed to the Guide for the Care and Use of Laboratory Animals published by the US National Institutes of Health (NIH Publication, 8th Edition, 2011). The animal care and experimental protocols were approved by Nanjing University’s Committee on Animal Care. All experiments conformed to international guidelines on the ethical use of animals.

Mice were randomly assigned to various analyses. Investigators performing histological analysis were blinded. Investigators involved in animal handling, sampling, and raw data collection were not blinded.

### HFD feeding protocol

A mouse obesity model was established through chronic feeding mice with a HFD (60% kcal from fat, D-12492, Research Diets, New Brunswick, NJ) for 14 weeks starting at the age of 5 weeks. Normal chow diet-fed WT littermates were maintained on diet with 6% kcal from fat. Food and water were provided ad libitum. Mice were housed and kept on a 12 h light/dark cycle at 23 ± 1 °C.

### Histological analysis and immunofluorescence staining

Hematoxylin/eosin (H&E) was performed on the paraffin-embedded WAT sections to evaluate the averaged adipocyte areas and histological changes. The adipocyte areas were measured in ten randomly areas of each sample using Cellsens Dimention 1.15 software (Olympus, Tokyo, Japan).

To investigate the expression of HSPA12A, immunofluorescence staining was performed on 4% PFA-fixed frozen WAT sections according to our previous method [34, 35]. Briefly, after incubation with the primary antibody for HSPA12A (#AB103030, 1:100, abcam) overnight at 4 °C, Cy3-conjugated or FITC-conjugated secondary antibody was applied to the sections to visualize the staining. Hoechst 33342 reagent was used to counterstain the nuclei. The staining was observed using a fluorescence microscope and quantified using Cellsens Dimention 1.15 software (Olympus, Tokyo, Japan).

### Quantitative real-time PCR

After total mRNA isolation and cDNA synthesis, PCR amplification was performed with SYBR Green PCR Master Mix (Roche). Quantitative real-time PCR were performed as described [36]. The primers used for PCR were listed in Table S1.

### Serum metabolic parameters

After fasting overnight, blood was drawn from humans and mice. Serum was separated for the analyses of metabolic parameters including FFA, LDL-C, HDL-C, CHOL, triglyceride (TG), and glucose using a Beckman Coulter AU5800 Chemistry System analyzer (Brea, CA).

### Glucose and insulin tolerance test

Glucose tolerance tests (GTTs) were performed by intraperitoneal injection of glucose (1.5 g/kg) to 18-week-old mice after a 16-h fast according to previous studies [37]. For insulin tolerance tests (ITTs), mice were intraperitoneal injected with regular human insulin (Eli Lilly & Company, Indianapolis, IN) at a dose of 1U/kg after a 6-h fast. Glucose was monitored in tail blood.

### Adenovirus construction

The adenoviral vector containing 3 flags-tagged coding region of mouse *Hspa12*a (NM_175199) was generated by GeneChem (Shanghai, China). The scheme of virus construction was shown in Figure S11.

### Cell culture, differentiation, and treatment

To prepare the stromal vascular fraction (SVF), inguinal WAT from 5-week-old mice were minced and digested with collagenase II (1.5 mg/mL). Digestions were stopped by adding ice-cold DMEM plus 10% FBS followed by centrifugation (1100×*g*) and filtration on prewet 40-μm cell strainers. SVFs were plated at 1 × 10^5^ cells in six-well plates or 2.5 × 10^4^ in 24-well plates and grown in DMEM supplied with 20% FBS. 3T3-L1 cells were from American Type Culture Collection and maintained in DMEM containing 10% FBS.

After confluence for 2 days, differentiation of primary SVF or 3T3-L1 cells were induced with 1 μM dexamethasone, 0.5 mM IBMX, and 5 μg/mL insulin for 2 days and then maintained in the medium containing only 5 μg/mL insulin for 2 days, followed by maintaining in regular medium for 2 days.

For PPARγ activation or inhibition, rosiglitazone (1μM) or GW9662 (20μM) was introduced to the cell cultures for the indicated durations according to previous studies [38].

For overexpressing HSPA12A (*Hspa12a*^*o/e*^), primary SVF or 3T3-L1 cells were infected with adenovirus that carrying *Hspa12a* expression sequence 2 days before confluency. The cells infected with empty adenovirus served as normal controls (NC).

### Differentiation capacity

Differential efficiency of adipocytes was evaluated using ORO or Nile red staining according to previous studies [39, 40]. Briefly, differentiated adipocytes in 24-well plates were fixed in 4% PFA (pH 7.4) for 30 min. For Nile red staining, the fixed cells were incubated with 0.1 μg/mL Nile red for 15 min. Images were observed and captured using a fluorescence microscope (magnification 200×). The relative fluorescence intensity was measured using a fluorometer (BioTek Synergy, Winooski, VT) at an excitation/emission wavelength of 543/598 nm and used as an indicator of lipid accumulation (surrogate for differentiation). For ORO staining, the fixed cells were incubated with ORO (2 mg/mL) for 30 min. After observation using a microscope (Zeiss Ltd., Germany), the stained ORO was extracted with isopropanol and quantified using a spectrophotometry at a wave length of 510 nm.

### Chromatin immunoprecipitation assay (ChIP)

Primary SVF with or without differentiation were fixed with formaldehyde at 37 °C for 10 min. The reaction was then stopped by the addition of 0.125 M glycine for 5 min. Cells were harvested, sonicated, and centrifuged (13,000 rpm). The soluble chromatin was precleared for 2 h with Protein A–Sepharose. Precleared chromatin was then incubated for 18 h with 2 μg of anti-mouse PPARγ antibody (#ab41928, abcam) or equal amount control mouse IgG antibody (#SC2025, SantaCruz) in the presence of BSA and salmon sperm DNA. Immune complexes were collected by incubation of 20 μL of Protein A–Sepharose beads for 2 h. Beads were extensively washed before reverse cross-linking. DNA was purified using a QiaQuick PCR purification kit (Qiagen) and subsequently analyzed by qPCR. The PCR products were also separated on 2% agarose gel. The amplification of promoter region containing the putative PPRE (−1096/−1087) was −1111 to −953 upstream of the transcriptional start site, and primers used were 5′-GGCTTTGGTAGCAGACCTCA-3′ and 5′-AACTTGGCATGGGAGGTTTA-3′.

### Immunoblotting and immunoprecipitation-immunoblotting analysis

Primary antibodies directly againt SCD1 (#2794, 1:1000), PPARγ (#2435, 1:1000) and C/EBPα (#8178, 1:1000) were all from Cell Signaling Technology (Beverly, MA); antibodies for against ACC (#BS1377, 1:1000), FABP4 (#BS6016, 1:1000) and GAPDH (#AP0063, 1:2000) were from Bioworld Technology (Louis Park, MN); anti-HSPA12A antibody (#AB103030, 1:1000) was abcam (Cambridge, MA). Protein was extracted from WAT or cells for immunoblotting according to our previous methods [34, 35]. To control for lane loading, the membranes were probed with anti-GAPDH antibody. The signals were quantified by scanning densitometry and the results from each experimental group were expressed as relative integrated intensities (compared with those of controls).

Immunoprecipitation-immunoblotting was performed according to previous methods [41]. The 3T3-L1 cells with or without differentiation were precipitated with anti-HSPA12A antibody. The cell lysates without immunoprecipitation served as positive controls (input), while the immunoprecipitates with normal IgG served as negative controls.

### Statistical analysis

Data represent as mean ± standard error. Groups were compared using Student’s two-tailed unpaired *t*-test, one-way or two-way ANOVA followed by Tukey’s test as a post-hoc test. No statistical methods were used to predetermine sample sizes. A *P* value of <0.05 was considered as significant.

## Supplementary information

Supplemental materials

Table S1

Supplemental data
