# Supplementary material for: HSPA12A is required for adipocyte differentiation and diet-induced obesity through a positive feedback regulation with PPARγ
Source: Cell Death Differ. 2019 Feb 11;26(11):2253–67. doi: 10.1038/s41418-019-0300-2 (PMC6888823; doi:10.1038/s41418-019-0300-2)
Supplement: Supplementary file 1 — Supplemental materials [file 41418_2019_300_MOESM1_ESM.docx]

**Supplementary Materials**

**Figure Legends**

**Figure S1. Histology of white fat tissues (WAT) from obese human and mice.**

1. Abdominal subcutaneous WAT (sWAT) were from obese patients (BMI > 35) and lean controls (BMI < 24). Hematoxylin/eosin (H&E) was performed on the paraffin-embedded WAT sections to evaluate the averaged adipocyte areas and histological changes. n = 6 mice/group. Scale bar = 50μm.
2. Inguinal WAT (iWAT) were collected from mice that fed with high-fat diet (HFD) or normal chow diet for 14 weeks starting at the age of 5 weeks. Hematoxylin/eosin (H&E) was performed on the paraffin-embedded WAT sections to evaluate the averaged adipocyte areas and histological changes. n = 6/group. Scale bar = 50μm.

**Figure S2. HFD increased *Hspa12a* mRNA expression in white adipose tissues of mice.**

Inguinal WAT were collected from WT mice that fed with HFD or chow diet for 14 weeks. The expression of *Hspa12a* mRNA was analyzed by real-time PCR. Data are mean ± SEM. ** *P <* 0.01 by Student’s two-tailed unpaired *t* test, n = 8/group.

.

**Figure S3. HSPA12A expression was absent in WAT of *Hspa12a*^-/-^ mice.**

Different WAT were collected from adult WT and *Hspa12a*^-/-^ mice. Immunoblotting was performed to evaluate HSPA12A protein expression. n = 10/group.

**Figure S4. *Hspa12a*^-/-^ mice showed no changes in body length and food intake.**

Mice at the age of 18-week were used in the measurements. Body length was examined from nose to tail root at a supine Position. Food intake was recorded consecutively for 7 days and expressed as the averaged intake per day. n = 6 – 8/group (body length) n = 4/group (food intake).

**Figure S5. Monoacylglycerol lipase expression.**

Inguinal WAT were collected from mice that fed with HFD or chow diet for 14 weeks. The expression of Monoacylglycerol lipase (*Mgl*) mRNA was analyzed by Real-time PCR. Data are mean ± SEM. two-way ANOVA followed by Tukey’s test. n = 6/group.

**Figure S6. Deficiency of HSPA12A suppressed expression of PPARγ target genes linking to adipocyte differentiation in mice.**

Inguinal WAT were collected from mice that fed with HFD or chow diet for 14 weeks. The expression of the indicated proteins was analyzed by Immunoblotting. Data are mean ± SEM, ** *P <* 0.01 and * *P <* 0.05 by two-way ANOVA followed by Tukey’s test. n = 6 – 7 mice /group.

**Figure S7. Effect of HSPA12A on the expression of PPARγ target genes linking to adipocyte differentiation in primary adipocytes.**

1. **HSPA12A deficiency decreased expression of PPARγ target genes**. Primary SVF were isolated from WT and *Hspa12a^-/-^* mice. Six days after differentiation, expression of the indicated proteins was examined by immunoblotting. Data are mean ± SEM, ** *P <* 0.01 by Student’s two-tailed unpaired *t* test. n = 8/group.
2. **HSPA12A overexpression increased expression of PPARγ target genes.** Primary SVF was isolated from WT mice and was overexpressed with HSPA12A (*Hspa12a^o/e^*). The SVF infected with empty-virus served as normal controls (NC). Six days after differentiation induction, the indicated protein expression was examined using immunoblotting. Data are mean ± SEM, ** *P <* 0.01 and * *P <* 0.05 by Student’s two-tailed unpaired *t* test. n = 4/group.

**Figure S8. Effect of PPARγ inhibition on *Hspa12a* mRNA expression in adipocytes.**

3T3-L1 preadipocytes were overexpressed with HSPA12A (*Hspa12a^o/e^*) by infected with adenovirus-carried *Hspa12a* expression sequence. 3T3-L1 preadipocytes infected with empty virus served as normal controls (NC). Six days after differentiation induction in the presence or absence of GW9662, the cells are harvested for *Hspsa12a* mRNA level analysis using real-time PCR. Data are mean ± SEM, ** *P <* 0.01 and * *P <* 0.05 by two-way ANOVA followed by Tukey’s test. n = 6/group.

**Figure S9. Immunoprecipitation-western blotting**

The 3T3-L1 cells with or without differentiation were lysed for precipitation with anti-HSPA12A antibody. The cell lysates without immunoprecipitation served as positive controls (input), while the immunoprecipitates of lysates with normal IgG served as negative controls. Immunoblotting against PPARγ or HSPA12A was performed subsequently.

**Figure S10. Strategy of generating *Hspa12a*^-/-^ mice.**

Exon 2-3 of *Hspa12a* gene was deleted using Cre-loxp recombinant system.

**Figure S11. Strategy of constructing the adenovirus containing *Hspa12a* expression sequence.**
