# Supplementary material for: HSPA12A is required for adipocyte differentiation and diet-induced obesity through a positive feedback regulation with PPARγ
Source: Cell Death Differ. 2019 Feb 11;26(11):2253–67. doi: 10.1038/s41418-019-0300-2 (PMC6888823; doi:10.1038/s41418-019-0300-2)
Supplement: Supplementary file 2 — Table S1 [file 41418_2019_300_MOESM2_ESM.docx]

**Table S1. Primers used in the experiments**

| **Gene name** | **Primers** |
| --- | --- |
| *Hspa12a for homo* | Forward GCTCCCACATCTGCATATTCAT |
|  | Reverse TTCTGAGACGTTGGAGTCAGT |
| *Gapdh for homo* | Forward ACAACTTTGGTATCGTGGAAGG |
|  | Reverse GCCATCACGCCACAGTTTC |
| *Hspa12a for mus* | Forward CACAGGGGTGAGTTGGTCTC |
|  | Reverse TGAGGAGCCTTCCAGGCTAT |
| *Pparq for mus* | Forward GAGCACTTCACAAGAAATTACC |
|  | Reverse GAACTCCATAGTGGAAGCCT |
| *Cebpa for mus* | Forward CAAGAACAGCAACGAGTACCG |
|  | Reverse AGGCGGTCATTGTCACTGGT |
| *Adipoq for mus* | Forward TGTTCCTCTTAATCCTGCCCA |
|  | Reverse CCAACCTGCACAAGTTCCCTT |
| *Cebpb for mus* | Forward AGAAGACGGTGGACAATGTGA |
|  | Reverse GTCAGCTCCAGCACCTTGTG |
| *Srebp-1c for mus* | Forward ACGGAGCCATGGATTGCACATTTG |
|  | Reverse AGGCTGTAGGATGGTGAGTGG |
| *Fasn for mus* | Forward TGGTGGTGTGGACATGGTCACAGA |
|  | Reverse CCGAAGCTGGGGGTCCATTGTGTG |
| *Scd1 for mus* | Forward AGCTGGTGATGTTCCAGAGG |
|  | Reverse TGAGCACCAGAGTGTATCGC |
| *Elovl6 for mus* | Forward TCCACGATTTCCCAGAGAAC |
|  | Reverse GAGCACCGAATATACTGAAGACG |
| *Acc for mus* | Forward CCCAGCAGAATAAAGCTACTTTGG |
|  | Reverse TCCTTTTGTGCAACTAGGAACGT |
| *Lipe for mus* | Forward CCAGCCTGAGGGCTTACTG |
|  | Reverse CTCCATTGACTGTGACATCTCG |
| *Atgl for mus* | Forward GAGACCAAGTGGAACATC |
|  | Reverse GTAGATGTGAGTGGCGTT |
| *Mgl for mus* | Forward TGGCCTGAAGCTGACAAGTA |
|  | Reverse AGGCCGATCCAACTAACCACAT |
| *Actin for mus* | Forward TGTTACCAACTGGGACGACA |
|  | Reverse TCTCAGCTGTGGTGGTGAAG |
| *Fabp4 for mus* | Forward AAGGTGAAGAGCATCATAACCCT |
|  | Reverse TCACGCCTTTCATAACACATTCC |
| *Cd36 for mus* | Forward ATGGGCTGTGATCGGAACTG |
|  | Reverse GTCTTCCCAATAAGCATGTCTCC |
| *Fabp4 for mus* | Forward AAGGTGAAGAGCATCATAACCCT |
|  | Reverse TCACGCCTTTCATAACACATTCC |
