# Supplementary material for: HSPA12A is required for adipocyte differentiation and diet-induced obesity through a positive feedback regulation with PPARγ
Source: Cell Death Differ. 2019 Feb 11;26(11):2253–67. doi: 10.1038/s41418-019-0300-2 (PMC6888823; doi:10.1038/s41418-019-0300-2)
Supplement: Supplementary file 3 — Supplemental data [file 41418_2019_300_MOESM3_ESM.pptx]

## Slide 1
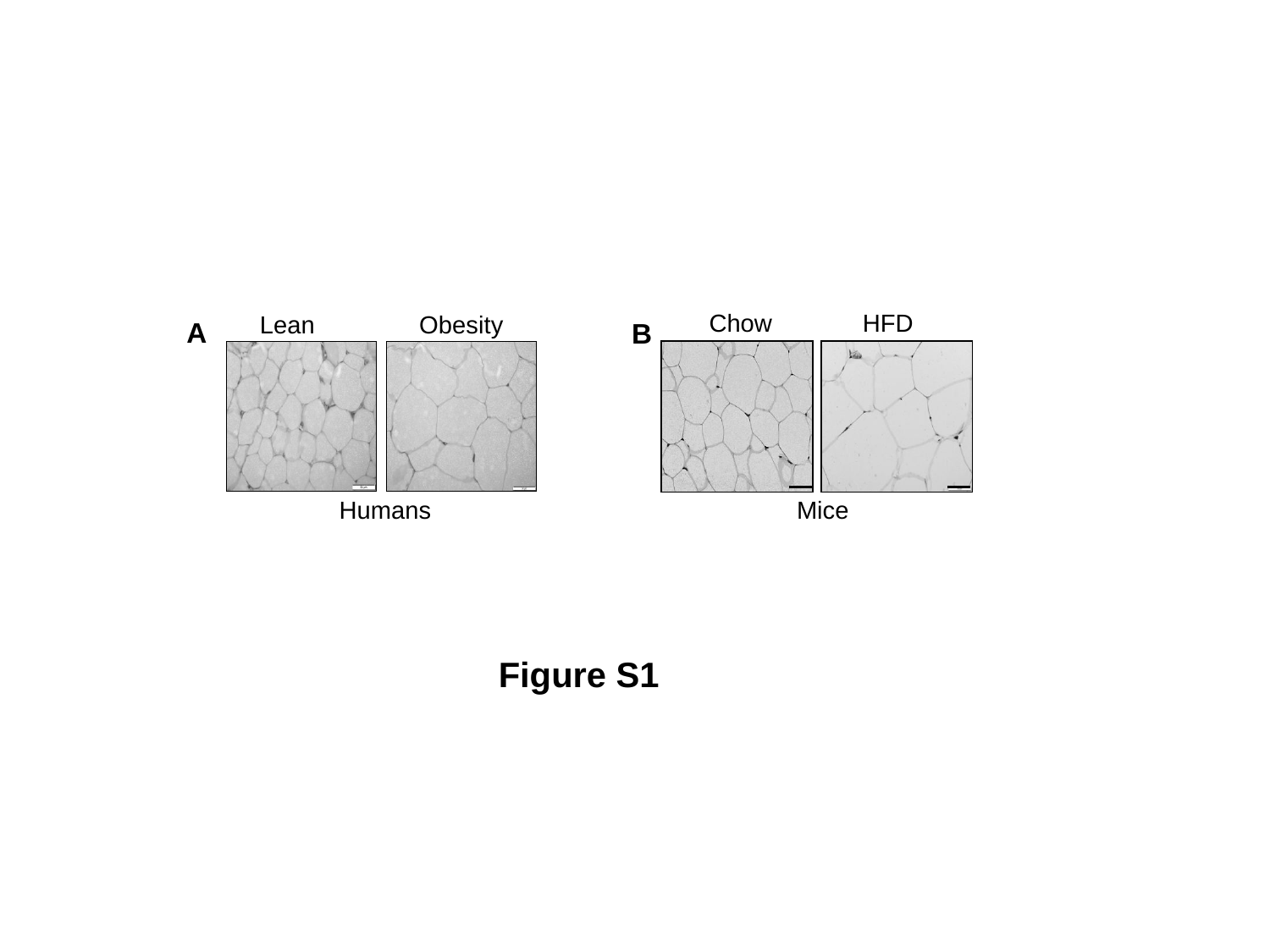

Chow HFD
B
Mice
Lean Obesity
A
Humans
Figure S1

## Slide 2
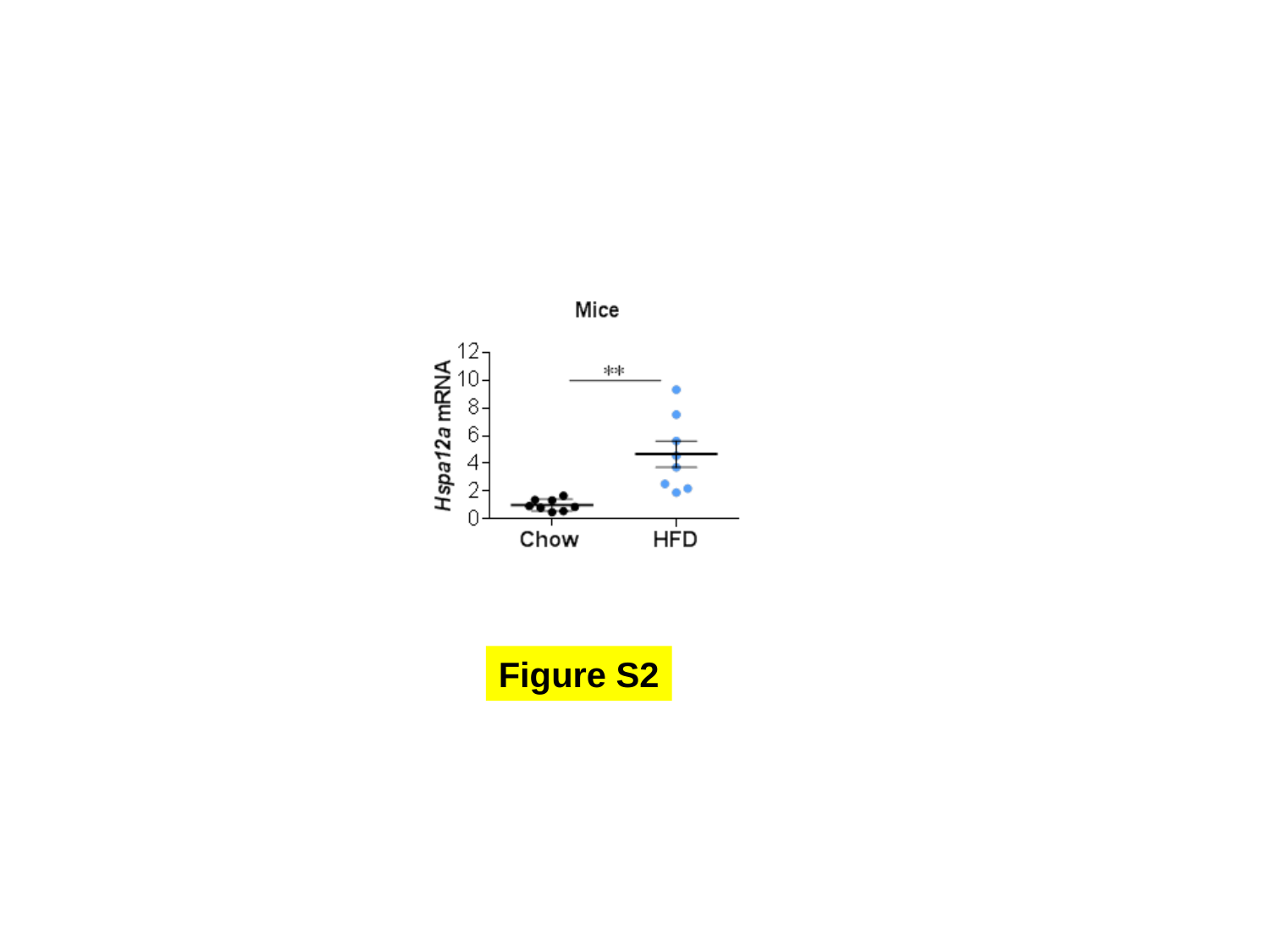

Figure S2

## Slide 3
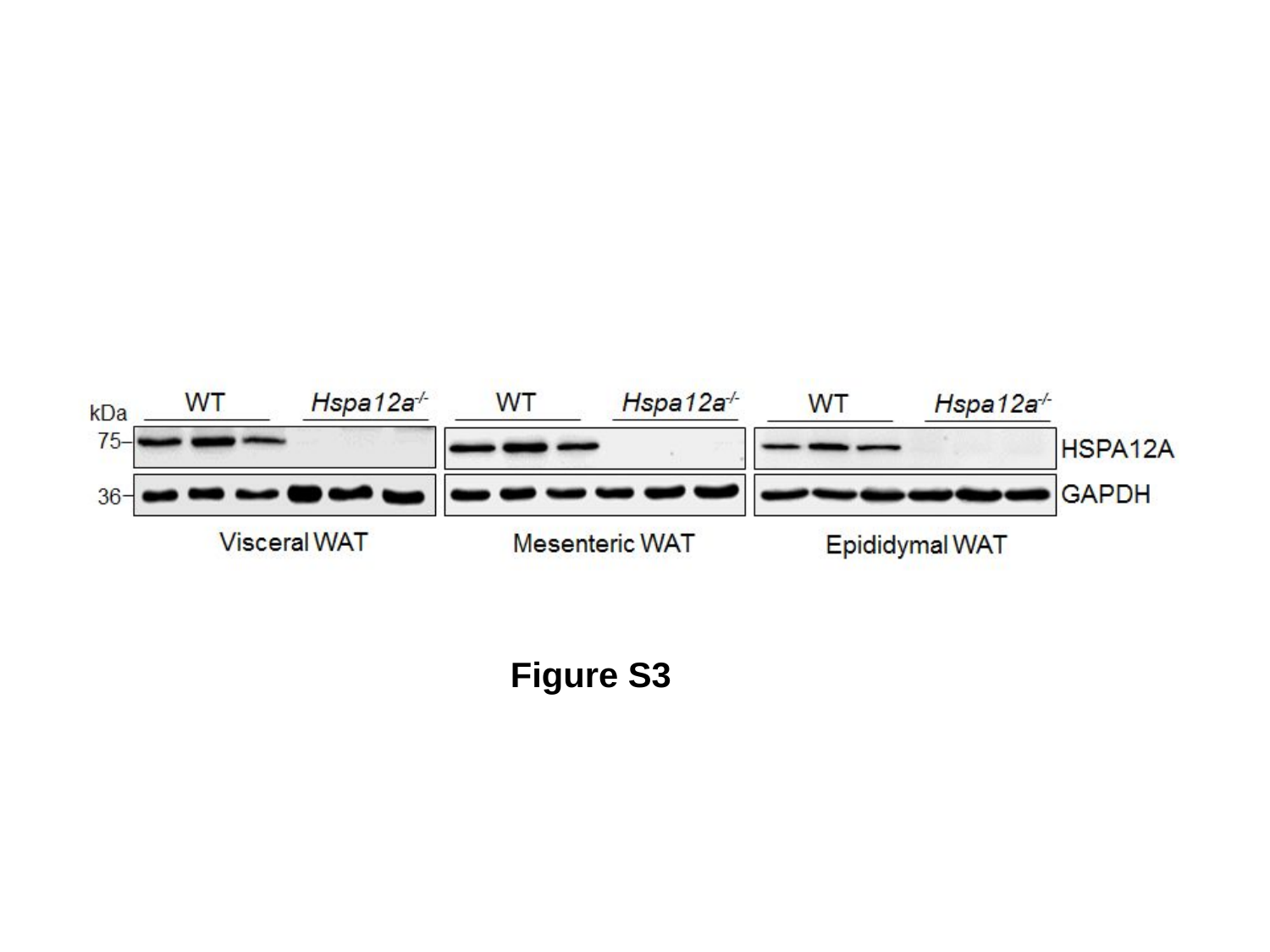

Figure S3

## Slide 4
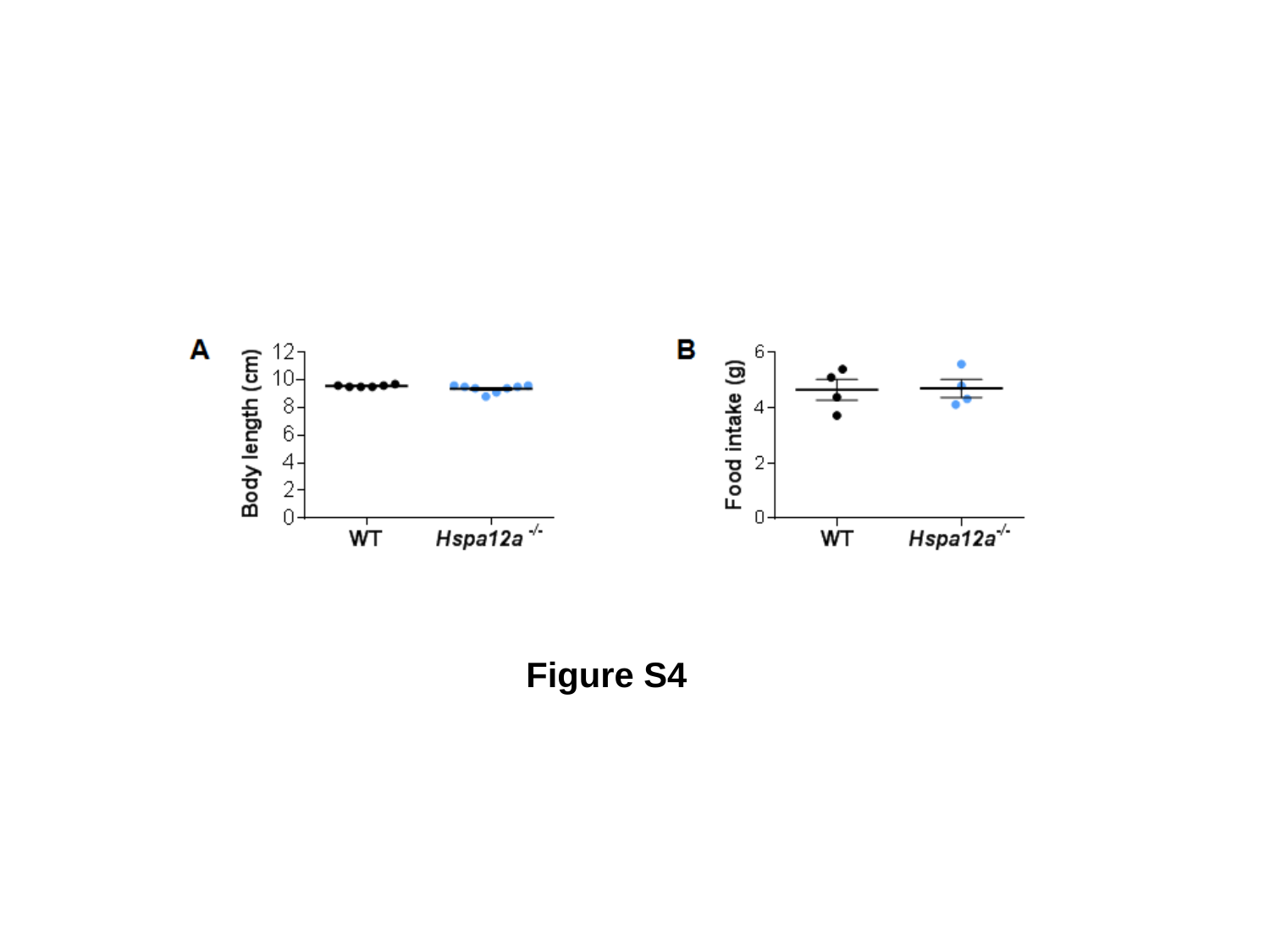

Figure S4

## Slide 5
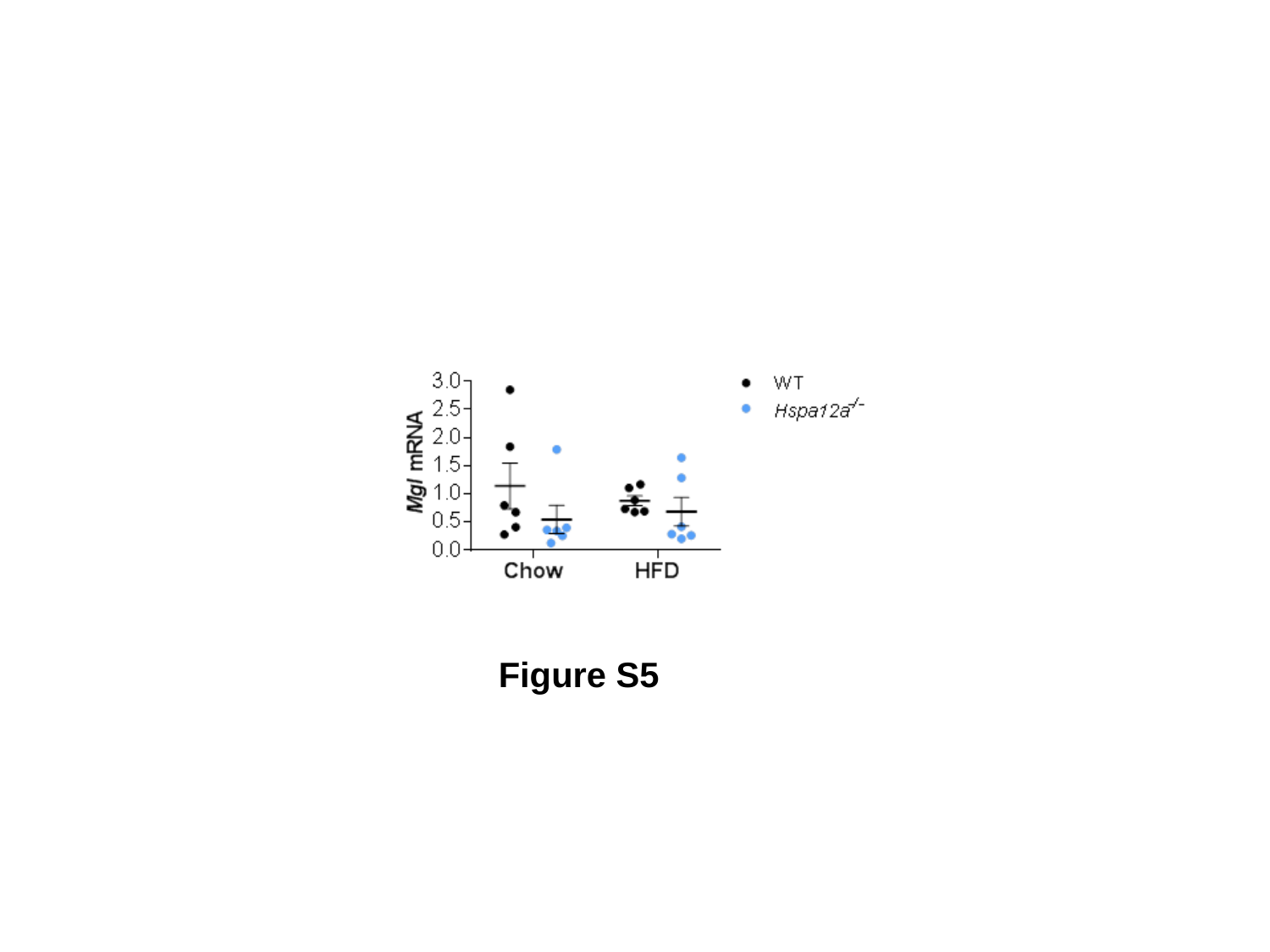

Figure S5

## Slide 6
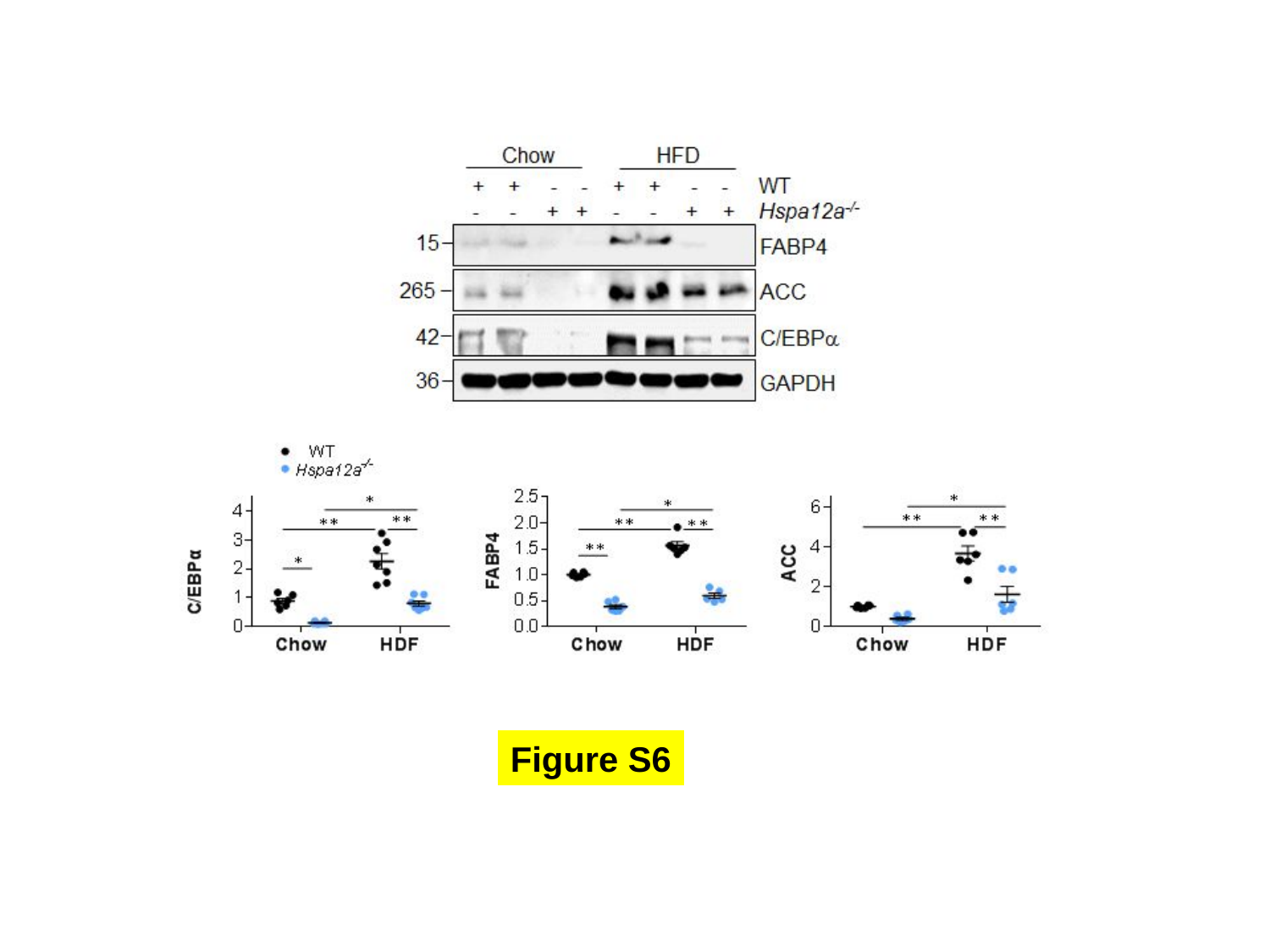

Figure S6

## Slide 7
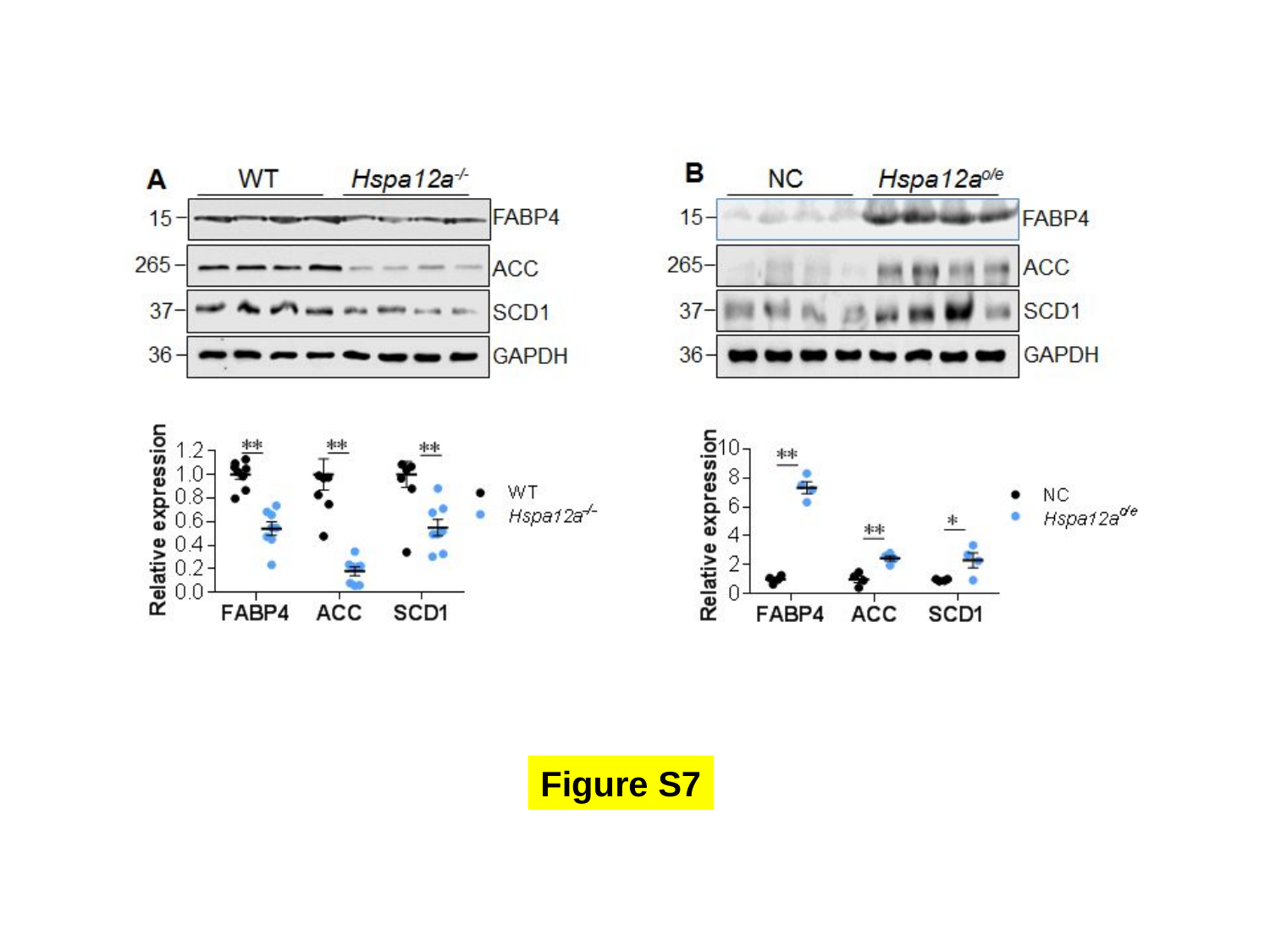

Figure S7

## Slide 8
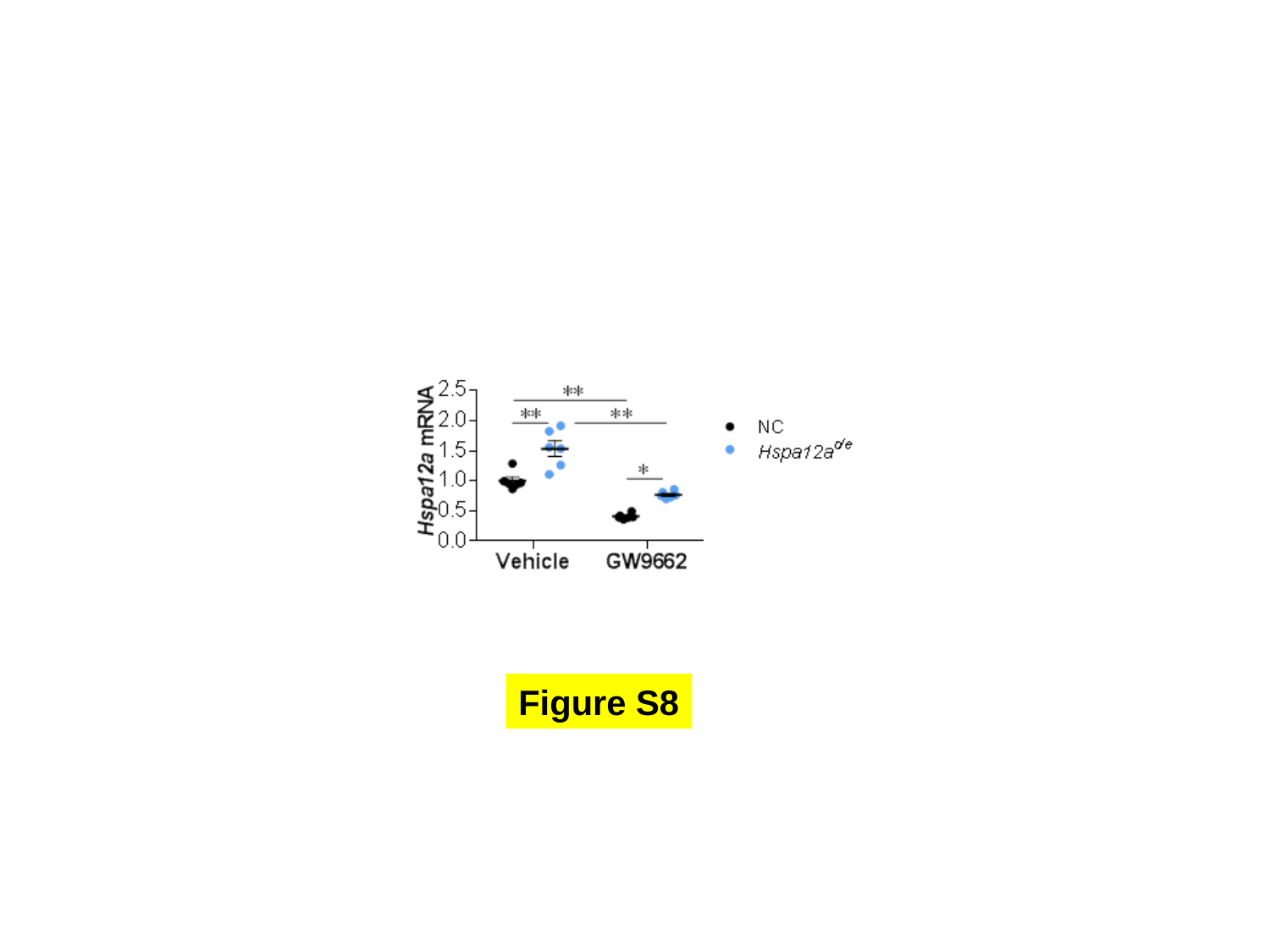

Figure S8

## Slide 9
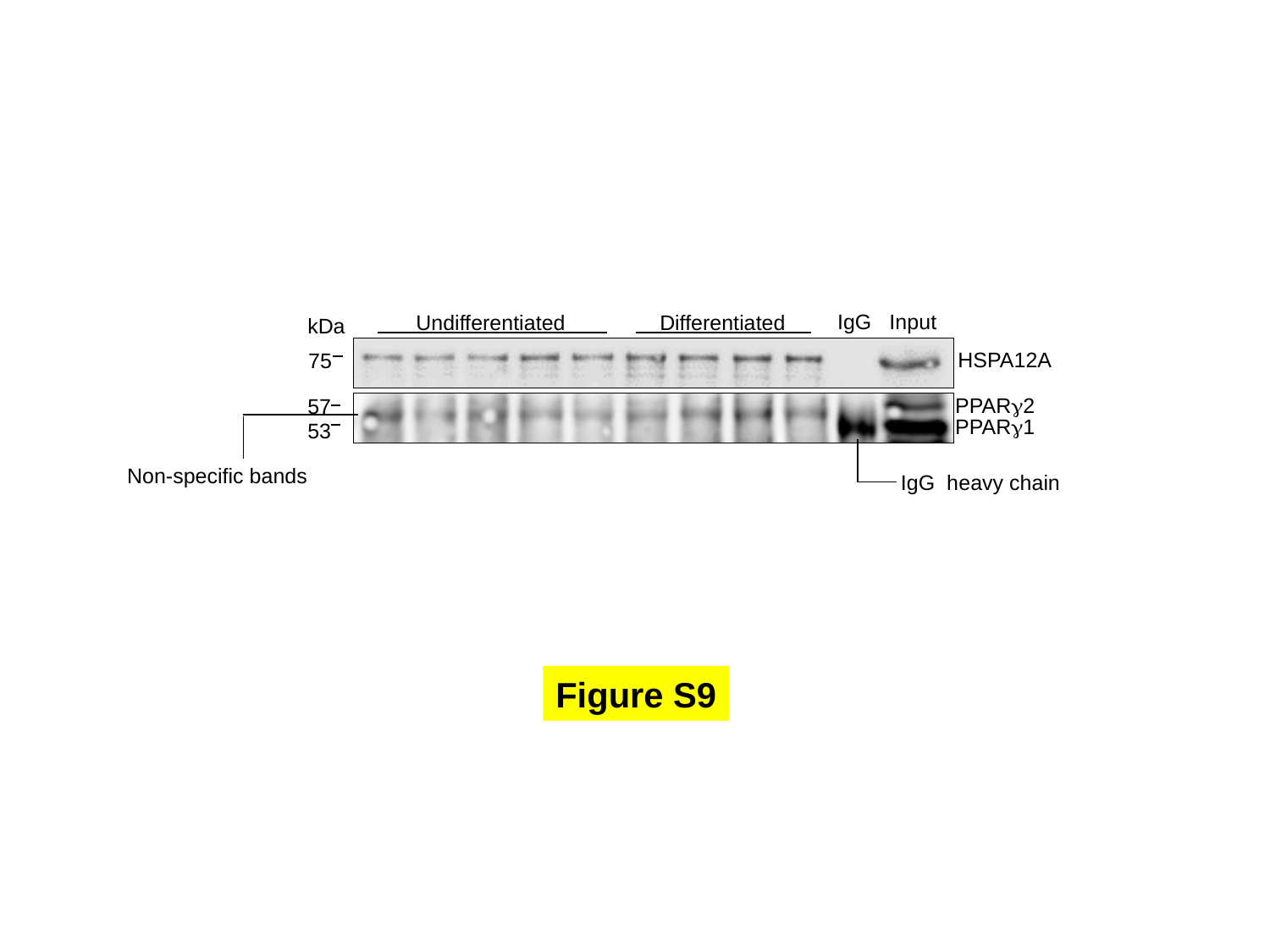

IgG Input
 Undifferentiated Differentiated
HSPA12A
PPARγ2
PPARγ1
kDa
75
57
53
Non-specific bands
IgG heavy chain
Figure S9

## Slide 10
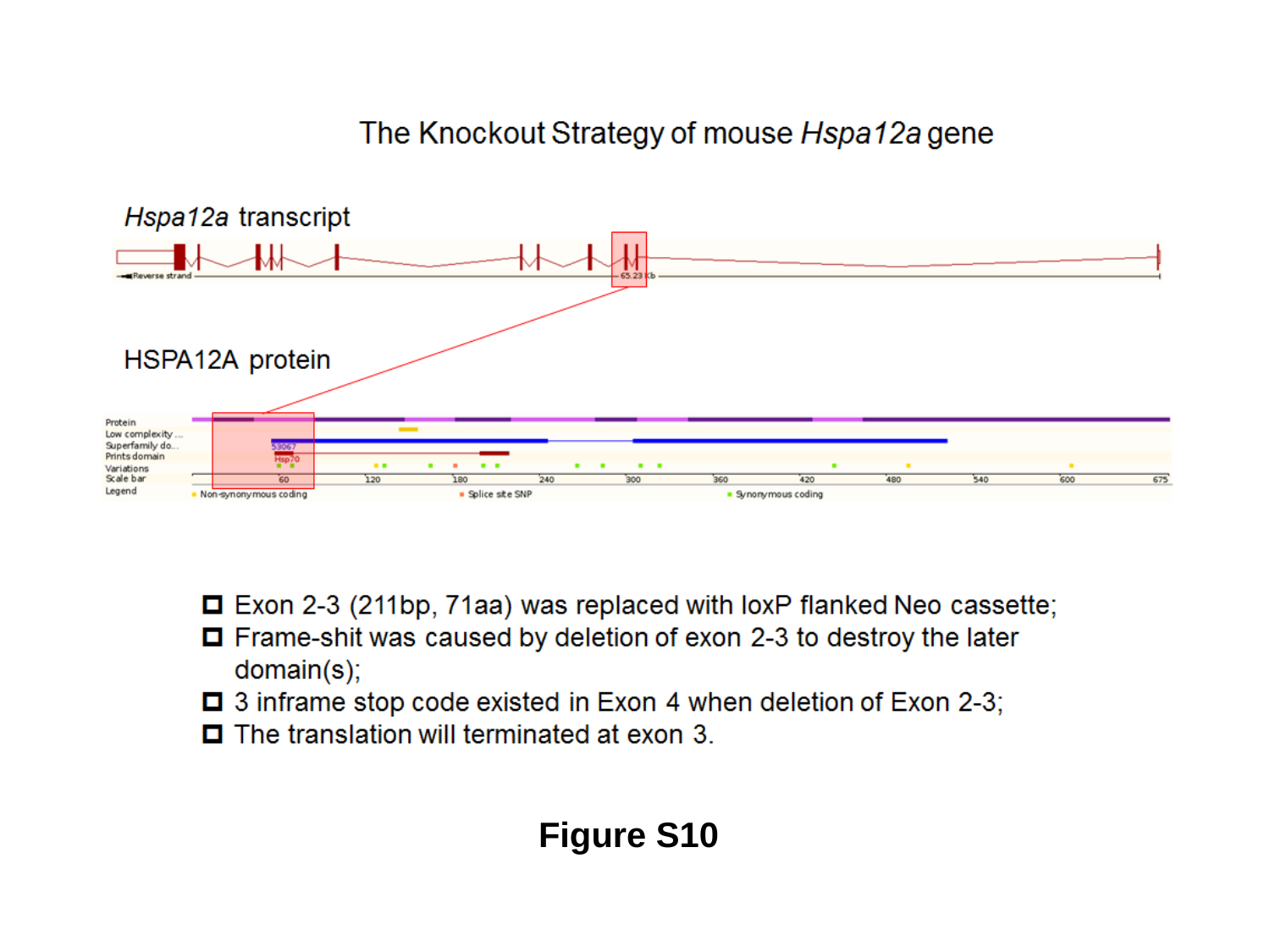

Figure S10

## Slide 11
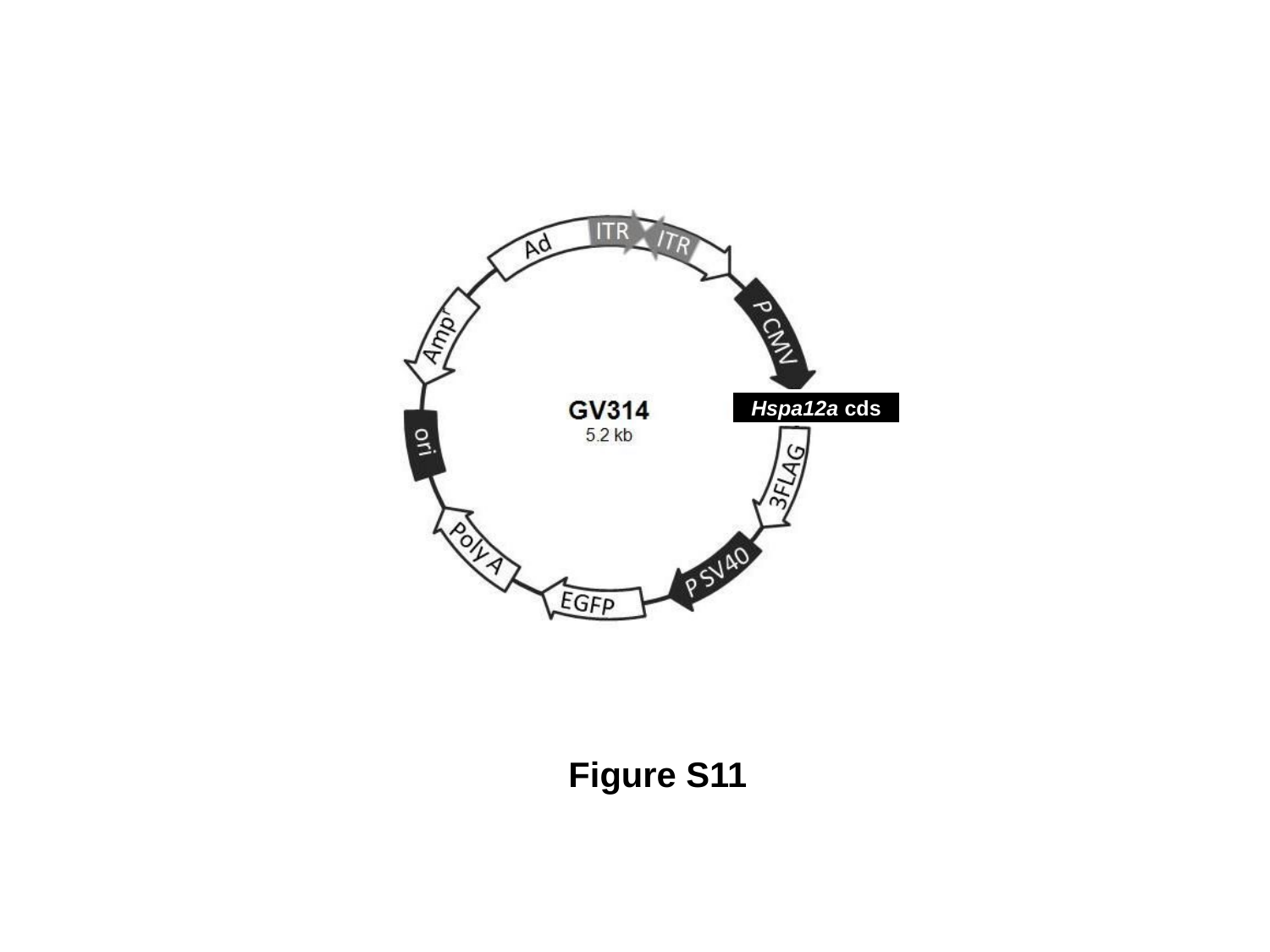

Hspa12a cds
Figure S11
